# Divergent interfaces and convergent outcome in the structural basis of HIV-2 Vif antagonism of APOBEC3H

**DOI:** 10.1038/s41467-026-78197-2

**Published:** 2026-10-07

**Authors:** Katarzyna A. Skorupka, Kazuhiro Matsuoka, Mayumi Imahashi, Yoshiyuki Yokomaku, Yasumasa Iwatani, Hiroshi Matsuo

**Affiliations:** 1 Cancer Innovation Laboratory, Frederick National Laboratory for Cancer Research, Frederick, MD 21702 USA; 2 Clinical Research Center, NHO Nagoya Medical Center, Nagoya, Aichi Japan; 3 Department of AIDS Research, Division of Basic Medicine, Nagoya University Graduate School of Medicine, Nagoya, Aichi Japan; 4 Department of Microbiology & Immunology, Hamamatsu University School of Medicine, Hamamatsu, Shizuoka Japan; 5 Present Address: Division of Host Defense Sciences, Omics Health Sciences, Department of Integrated Health Sciences, Nagoya University Graduate School of Medicine, Nagoya, Japan

**Keywords:** Cryoelectron microscopy, HIV infections, Immune evasion, Restriction factors

## Abstract

Primate lentiviruses counteract antiviral APOBEC3 (A3) cytidine deaminases through virion infectivity factor (Vif), which recruits A3 proteins to a Cullin–RING E3 ubiquitin ligase for proteasomal degradation. While HIV-1 Vif is well characterized, HIV-2 Vif remains poorly defined due to divergent sequences. Here we report a 3.08 Å cryo-EM structure of HIV-2 Vif bound to chimpanzee A3H (cpzA3H) and E3 ligase components. HIV-2 Vif adopts a conserved global fold but engages cpzA3H through a binding interface distinct from HIV-1 Vif, achieving target recognition through numerous weak contacts distributed across an extended interface. The structure reveals a direct CBFβ–cpzA3H interface absent in HIV-1 Vif-A3H complexes, establishing CBFβ as a bivalent adaptor that actively contributes to substrate recruitment. Despite these structural differences, lysine 50 of cpzA3H is the shared ubiquitination target of both HIV-1- and HIV-2 Vif-induced degradation, revealing convergent function achieved through divergent structural solutions.

## Abstract (web summary)

HIV-2 Vif antagonizes host APOBEC3 restriction factors, but its mechanism remains poorly defined. Here, authors report the cryoEM structure of HIV-2 Vif bound to APOBEC3H, revealing a CBFβ-mediated interface distinct from HIV-1 Vif.

## Introduction

APOBEC3 (A3) cytidine deaminases constitute a key arm of intrinsic immunity that restricts retroviral replication by deaminating cytosines in nascent viral cDNA, leading to G-to-A hypermutation and loss of infectivity^1–9^. Among the seven human A3 proteins, APOBEC3H (A3H) is particularly notable for its potent antiviral activity and extensive polymorphism, which modulates A3H stability, subcellular localization, and susceptibility to viral antagonism^10–18^. A distinctive feature of A3H among the A3 family is its ability to form an obligate homodimer bridged by a short double-stranded RNA (dsRNA) molecule, a mode of dimerization that is critical for protein stability, cytoplasmic localization, virion packaging, and antiviral activity^19–21^. This dsRNA-mediated dimer architecture, in which two A3H protomers are held in proximity without direct protein-protein contacts, is structurally distinct from other A3 family members. Additionally, the A3H dimer architecture has important consequences for how virion infectivity factor (Vif) recognizes and antagonizes A3H, compared to other A3s.

Vif is a primate lentiviral protein that has evolved to antagonize host APOBEC3 (A3) proteins, including A3H, by recruiting them to the cellular Cullin-RING E3 ubiquitin ligase machinery and facilitating their proteasomal degradation^1–7,14,22–27^. HIV-1 Vif is the best-characterized A3 antagonist and recruits a Cullin 5 (CUL5)–RBX2–Elongin B/Elongin C (ELOB/C) complex, requiring the noncanonical cofactor core-binding factor β (CBFβ) for structural stability and activity, as demonstrated by two landmark independent studies^28,29^. Subsequent structural studies have shown that HIV-1 Vif positions A3 substrates for polyubiquitination through a combination of direct protein-protein contacts and, in some cases, RNA-mediated interfaces^30–35^ that vary significantly depending on the specific A3 family member present. For example, the structure of A3H recruited to HIV-1 Vif-CBFβ–ELOB/C–CUL5 complex revealed direct contacts between A3H and Vif that bury a modest 700 Å² of surface area^34,35^, whereas A3F engages a surface formed by Vif–CBFβ of much larger size (~1000 Å²)^30^. On the other hand, interactions between A3G and Vif are primarily mediated through a single-stranded RNA (ssRNA) chain wedged between the two proteins^31–33^. These examples illustrate how HIV-1 Vif adapts to intercept different members of the A3 family and showcase a remarkable structural and functional versatility of this lentiviral factor.

In contrast, HIV-2 Vif remains less well characterized. HIV-2 derives from simian immunodeficiency virus infecting sooty mangabeys (SIVsmm) and is highly divergent from HIV-1 Vif, which originated from SIV lineages infecting chimpanzees (SIVcpz) and gorillas^36^. This divergence reflects adaptation to a different primate reservoir and results in marked differences in Vif sequence and APOBEC3 substrate specificity^37,38^, suggesting that HIV-1 and HIV-2 Vif proteins have evolved independent solutions to the same host-restriction pressure. However, given the understudied nature of HIV-2 Vif, these questions remain open. Beyond differences between the Vif proteins, HIV-1 and HIV-2 infections themselves are markedly different, with HIV-2 typically associated with lower viral loads, slower disease progression, and a longer asymptomatic period compared to HIV-1^39^. But whether differences in pathogenicity between HIV-1 and HIV-2 are influenced, even in part, by variation in APOBEC3 restriction and Vif-mediated antagonism remains unclear.

Here, we present the 3.08 Å-resolution structure of HIV-2 Vif (strain NMC716) in complex with chimpanzee A3H (cpzA3H), CBFβ, and ELOB/C, thereby addressing these knowledge gaps. The structure reveals the molecular basis of HIV-2 Vif–A3H recognition and uncovers a distinct, direct CBFβ–A3H interface. Structural and sequence comparison with the HIV-1 Vif–cpzA3H complex demonstrates that, despite a conserved Vif fold, the A3H-binding surfaces of HIV-1 and HIV-2 Vif are almost entirely non-overlapping, providing direct structural evidence for convergent evolution of A3H antagonism across two independently transmitted human retroviruses. Structural modeling of the complete HIV-2 Vif–cpzA3H–CBFβ–CUL5 E3 ligase assembly, together with mass spectrometry-based identification of ubiquitination sites, defines the spatial organization of the ubiquitination machinery and illuminates the mechanism of ubiquitin transfer initiation. Together, these findings illuminate how HIV-2 Vif has adapted to recognize primate A3H proteins and provide a structural framework for understanding differences in A3 antagonism across lentiviral lineages.

## Results

### The architecture of the HIV-2 Vif–cpzA3H–CBFβ–EloBC complex

To elucidate the molecular basis of HIV-2 Vif-mediated antagonism of the cpzA3H restriction factor, we solved a cryo-EM structure of the cpzA3H bound to HIV-2 Vif (CRF01_AB, strain NMC716)^40^ / core-binding factor subunit β (CBFβ) / Elongin B / Elongin C (ELOB/C) complex (hereafter referred to as the V2CBC complex). The cpzA3H–V2CBC complex was formed by mixing equimolar amounts of cpzA3H dimer (formed upon binding a dsRNA) and the V2CBC complex using multi-step process to ensure high quality and integrity of the final cpzA3H–V2CBC complex (Supplementary Fig. 1a-f).

However, the purified cpzA3H–V2CBC complex exhibited pronounced preferred orientation on cryo-EM grids. To overcome this limitation, we supplemented the cpzA3H–V2CBC complex with N-terminal CUL5 (nCul5) at an equimolar ratio (Supplementary Fig. 1g), which resulted in improved cryo-EM data (Supplementary Fig. 2). This sample yielded a reconstruction with a global resolution of 3.12 Å as assessed by gold-standard Fourier shell correlation (GSFSC), with the corrected masked map resolving to 3.08 Å (Supplementary Fig. 3). Further post-processing using the EMready2 neural network algorithm^41^ enabled more precise placement of amino acid side chains within the EM map (Supplementary Fig. 4). Local resolution ranged from 2.6 to 3.8 Å across the complex, with the highest resolution observed at the cpzA3H–Vif interface and lower resolution in more peripheral regions, such as the distal protomer of the cpzA3H dimer (cpzA3H_B_) and ELOB/C (Supplementary Fig. 3). Additionally, although nCul5 was included during sample preparation, it was not observed in the final reconstruction. In contrast to the dimeric cpzA3H–VCBC complex observed in our previous study^35^, the cpzA3H–V2CBC complex is monomeric, as determined by size-exclusion chromatography and confirmed by cryo-EM structural analysis.

**Fig. 1:**
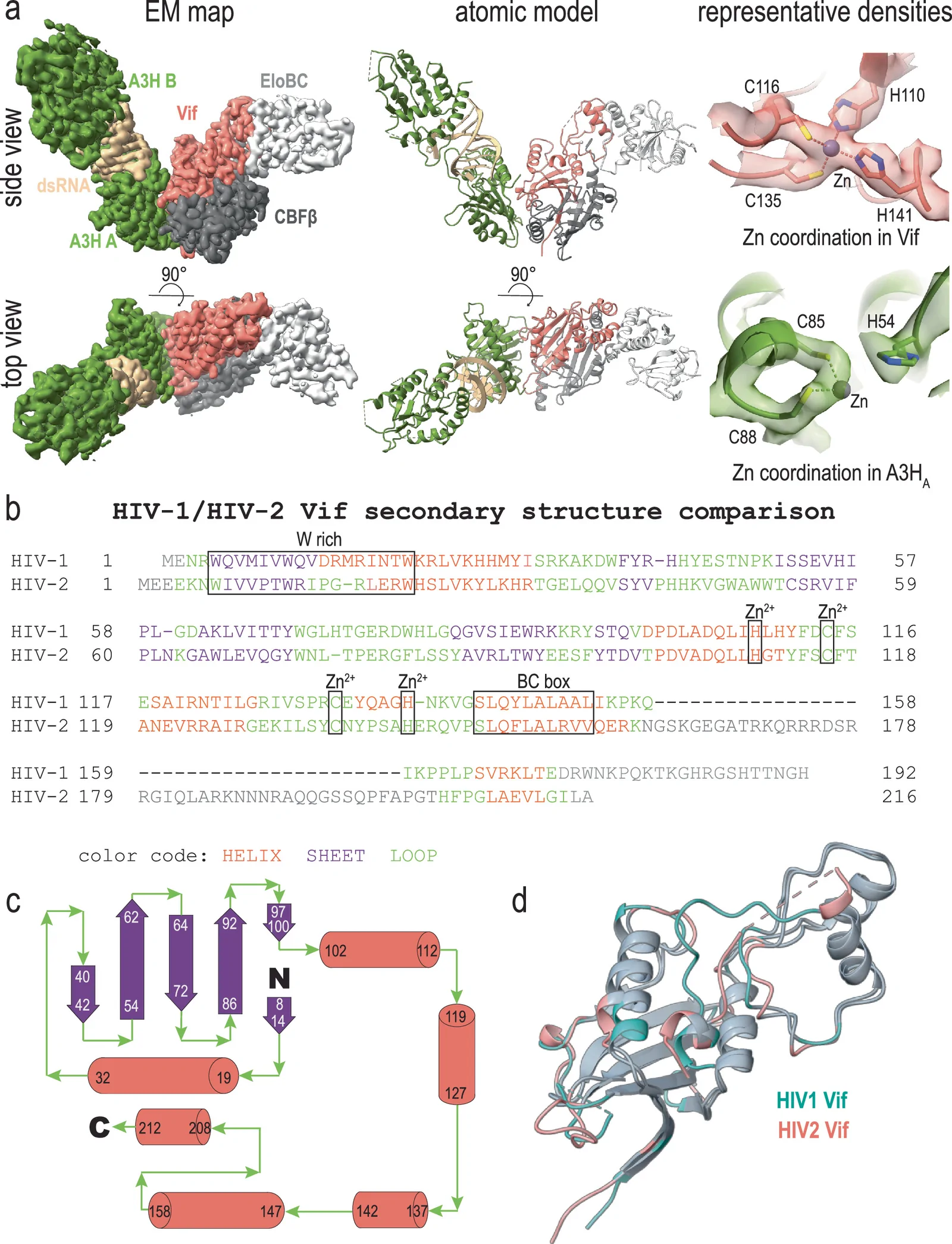
Cryo-EM structure of cpzA3H in complex with HIV-2 Vif–CBFβ–EloBC. **a** Cryo-EM reconstruction and atomic model of the cpzA3H–V2CBC complex. Left panels show segmented EM density map in side view (top) and top view (bottom), with individual components colored as follows: A3H subunit A and B (A3H A, A3H B, olive green), Vif (salmon pink), double-stranded RNA (dsRNA, wheat), ElonginB/C (EloBC, light gray), and CBFβ (dark gray). Middle panels show the corresponding atomic model in the same orientations. Right panels show representative cryo-EM densities at the zinc coordination sites: the HCCH zinc-binding motif in Vif (top right), coordinated by residues C116, H110, C135, and H141, and the zinc coordination site in A3H_A_ (bottom right), involving residues H54, C85, and C88. The zinc ion is shown as a gray sphere. **b** Structure-based sequence alignment of HIV-1 and HIV-2 Vif. Secondary structure elements are color-coded: helices (orange), β-strands (purple), and loops (green). Functionally important regions are indicated, including the tryptophan-rich (W rich) motif, zinc-coordinating residues (Zn²⁺), and the BC box. Dashes indicate gaps in the alignment. **c** Topology diagram of HIV-2 Vif illustrating the arrangement of secondary structure elements, with helices shown as rounded rectangles (orange) and β-strands as arrows (purple), connected by loops (green). The N- and C-termini are labeled. Numbers indicate residue positions. **d** Structural superposition of HIV-1 Vif (PDB: 9E93, teal) and HIV-2 Vif (this study, salmon) atomic models, highlighting conserved fold architecture (gray) alongside regions of structural divergence, particularly in loop regions and the C-terminal domain.

The overall architecture of the complex reveals an asymmetric assembly in which two cpzA3H molecules (A3H_A_ and A3H_B_) are bridged by dsRNA (Fig. 1a), consistent with the known RNA-dependent dimerization of cpzA3H (PDB:5z98)^21^. The V2CBC complex adopts a crescent-shaped architecture, similar to previously described structures of HIV-1 VCBC (PDB:4N9F)^42^. Despite limited sequence identity between HIV-1 and HIV-2 Vif proteins (Fig. 1b), structural superposition of HIV-2 Vif (NMC716, this study) onto the previously reported HIV-1 Vif structure in complex with cpzA3H (PDB:9E93)^35^ reveals a highly conserved overall fold, with an RMSD of 1.04 Å (Fig. 1c, d). Both proteins share a common secondary structure topology comprising a mixed α/β architecture, including a conserved arrangement of β-strands and α-helices that form the core scaffold (Fig. 1c).

To identify functionally important residues in HIV-2 Vif, we performed HiBiT-based degradation assays^43–45^ combined with systematic alanine scanning and monitored cpzA3H degradation (Supplementary Fig. 5a and b). These analyses demonstrated that Zn²⁺-coordinating residues (H110, C116, C135, and H141) are essential for Vif function, consistent with their roles in stabilizing the Vif structure and mediating recruitment of CUL5. In addition, BC-box residues L148, F150, and L153 contributed to activity, consistent with their established role in EloB/C recruitment. Alanine substitution of N-terminal tryptophan residues resulted in a marked reduction in cpzA3H degradation. W7 engages CBFβ I55 and V86, and W13 engages CBFβ L64 and Vif V87 and L89, through hydrophobic interactions essential for intermolecular β-sheet formation (Supplementary Fig. 5c). W22 contributes to a hydrophobic core stabilizing α-helix α1 and α-helix α6 on the β-sheets of HIV-2 Vif. Together, these data establish the high-resolution structure of HIV-2 Vif and provide a structural framework for understanding how HIV-2 Vif counteracts A3H-mediated restriction. Furthermore, our results indicate that, despite pronounced sequence divergence—HIV-1 NL4-3 and HIV-2 NMC716 Vif share only 22.9% identity and 37.2% similarity—these proteins converge on a conserved overall fold, including within the VCBC complex. Despite these similarities, we observed that A3H and HIV-2 Vif engage via distinct interfaces, which we describe in more detail below.

### Molecular architecture of the HIV-2 Vif–cpzA3H binding interface

**Fig. 2:**
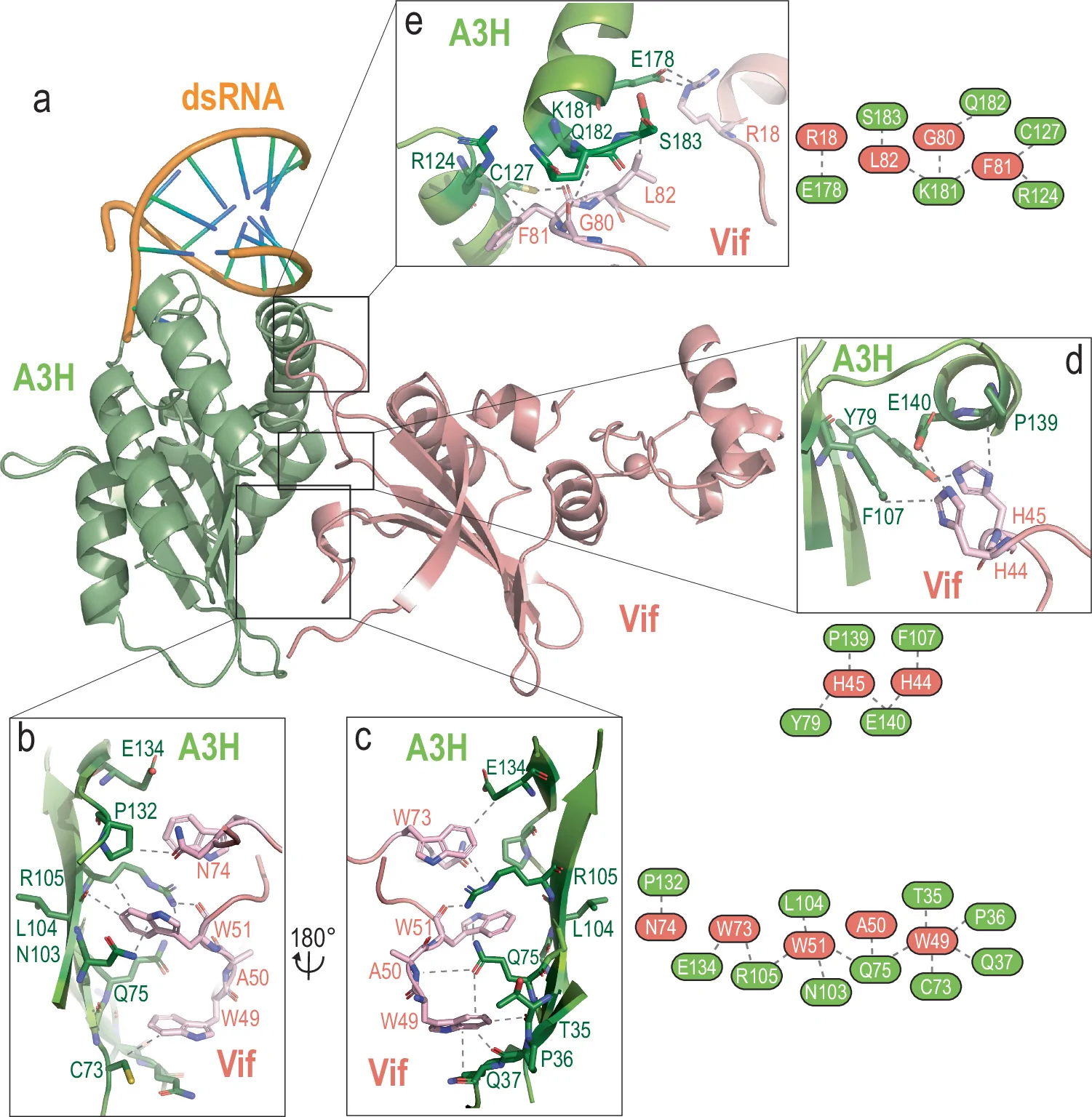
Molecular interfaces between HIV-2 Vif and cpzA3H. **a** Atomic model of the HIV-2 Vif (salmon)–cpzA3H (green)–dsRNA (yellow) complex shown in cartoon representation. The interaction surface was subdivided into three spatially distinct sub-interfaces, highlighted by boxed regions. **b–e** Close-up views of the three sub-interfaces highlighting key interacting residues, with accompanying schematics of cpzA3H–HIV-2 Vif contacts. cpzA3H residues are shown in green and Vif residues in salmon; dashed lines indicate potential hydrogen bonds, van der Waals interactions, and salt bridges. **b, c** Sub-interface 1, shown from two orientations related by a 180° rotation. Vif residues W49, A50, and W51 (loop 3), together with W73 and N74 (loop 5), engage cpzA3H residues on β1 (T35, P36, Q37), β3 (C73, Q75), β4 (N103, L104, R105), and β5 (P132, E134). These interactions form a hydrophobic cluster that packs against the β-sheet surface of cpzA3H and include a hydrogen bond between the backbone carbonyl of W51 and the guanidinium group of R105. **d** Sub-interface 2. Vif residues H44 and H45 contact cpzA3H residues Y79 (β3), F107 (β4), and P139 and E140 (α5). H44 is positioned to interact with the carboxylate group of E140, while additional contacts are consistent with local packing and weaker polar interactions. **e** Sub-interface 3. Vif residues R18 (loop 1) and G80, F81, and L82 (loop 5) engage cpzA3H residues R124 and C127 (α4), and E178, K181, Q182, and S183 (α6). A salt bridge between R18 and E178 is observed. The accompanying schematic summarizes the network of contacts at this interface.

The cryo-EM structure reveals an extensive interaction surface between HIV-2 Vif and cpzA3H, with a buried surface area of approximately 845 Å². The interface is stabilized by a combination of electrostatic, hydrogen bonding, and hydrophobic interactions, primarily involving flexible loop regions of Vif, including loop 3 (between β2 and β3) and loop 5 (between β4 and β5). To facilitate structural interpretation, the interface can be subdivided into three spatially distinct sub-interfaces (Fig. 2a), each contributing a network of intermolecular contacts.

The first sub-interface is formed by Vif residues W49, A50, and W51 located on loop 3, and W73 and N74 on loop 5. These residues engage cpzA3H residues T35, Q37, and P36 on β1; C73 and Q75 on β3; N103, L104, and R105 on β4; and P132 and E134 on β5 (Fig. 2b, c). The tryptophan residues at positions 49, 51, and 73 of Vif form a hydrophobic cluster that packs against the β-sheet surface of cpzA3H (Fig. 2b, c). Across the interface, a limited number of well-defined polar interactions are embedded within a broader network of hydrophobic and van der Waals contacts. In particular, a strong hydrogen bond is observed between the backbone carbonyl of W51 in Vif and the guanidinium group of R105 on cpzA3H, while additional contacts involving Vif W51 and neighboring residues to R105 on cpzA3H (*e.g*., L104 carbonyl and N103 amide) are consistent with weaker hydrogen bonding interactions. Aromatic residues, including W49, W51, W73, and F81 of Vif, contribute extensively to interface packing through van der Waals interactions with backbone carbonyls and side chains. W73 of Vif is positioned near the R105 guanidinium group of cpzA3H, consistent with a potential cation–π interaction, although the geometry suggests this interaction is modest.

A second sub-interface involves Vif residues H44 and H45 on loop 3, which contact cpzA3H residues Y79 (β3), F107 (β4), and E140 and P139 (α5) (Fig. 2d). H44 of Vif is positioned to form a hydrogen bond or electrostatic interaction with the E140 carboxylate of cpzA3H. Several contacts, involving H45 of Vif with the backbone carbonyl of P139 and the hydroxyl group of Y79 on cpzA3H, occur at distances ≥3.5 Å and are therefore best interpreted as contributing to local packing rather than forming strong, directional hydrogen bonds. The two HIV-2 Vif histidine residues (H44 and H45) are conserved in HIV-1 (H42 and H43) and, although they do not directly contact cpzA3H, they are critical for RNA-mediated bridging interactions that stabilize the A3G–HIV-1 Vif interface^31–33^.

A third, more distal contact region is mediated by Vif residues R18 on loop 1 and G80, F81, and L82 on loop 5, which engage cpzA3H residues R124 and C127 on α4, as well as E178, K181, Q182, and S183 at the end of α6 (Fig. 2e). A prominent electrostatic interaction is observed between Vif R18 and cpzA3H E178, consistent with a salt bridge that likely contributes to complex stability. Prior studies have not attributed a discrete, residue-specific mechanistic role to positions 182 and 183 of A3H; however, these residues define the C-terminal splice variants (SV182 and SV183) of human A3H, which differ by a single terminal residue and exhibit haplotype-dependent differences in antiviral activity^13^. In the context of our structure, these observations suggest that the functional differences between SV182 and SV183 likely arise from modulation of α6-mediated engagement with Vif.

Overall, the HIV-2 Vif–cpzA3H interface is characterized by a small number of key electrostatic and hydrogen bonding interactions supported by an extensive network of hydrophobic and van der Waals contacts that collectively stabilize the complex.

### Biochemical validation of HIV-2 Vif–cpzA3H contacts

**Fig. 3:**
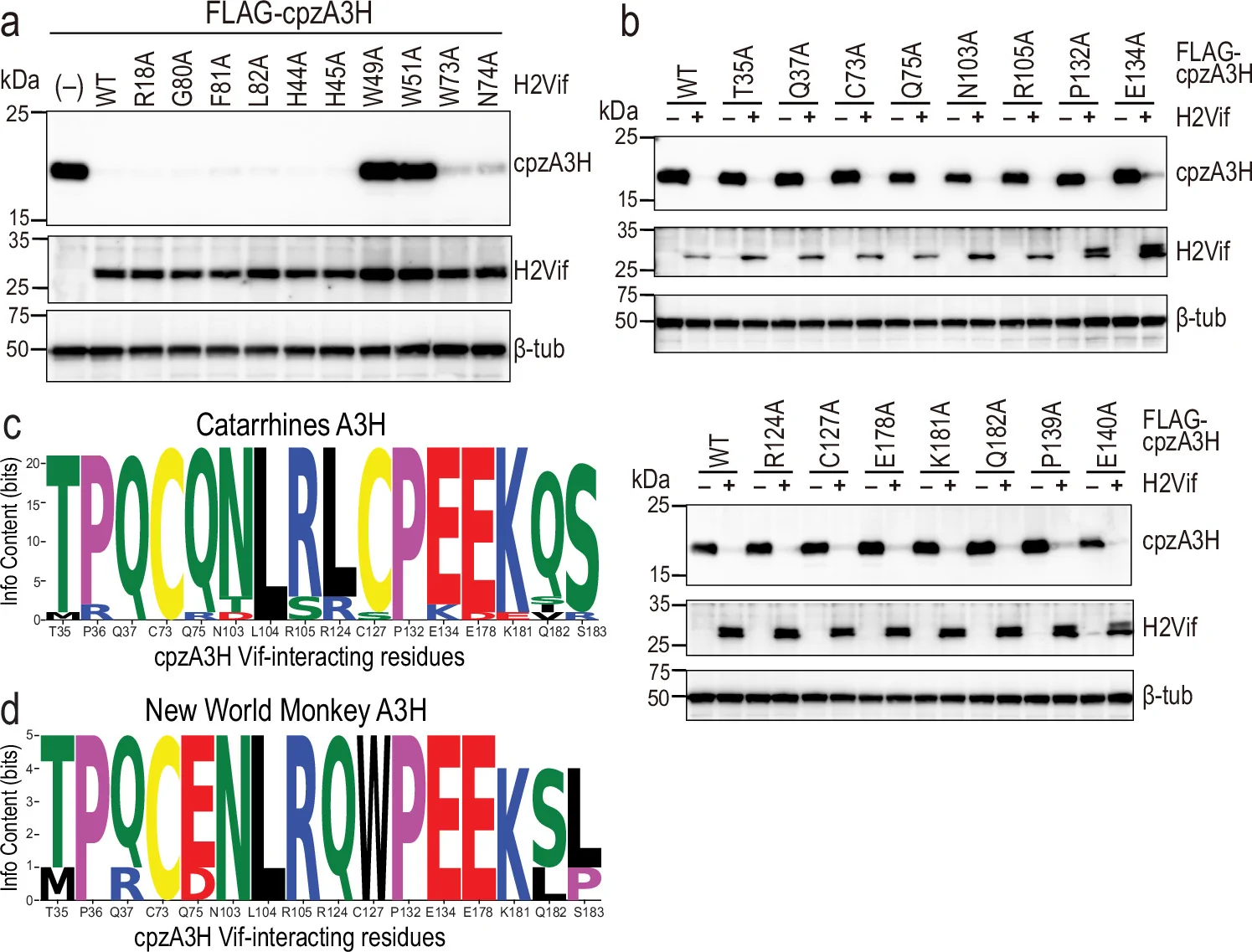
Mutational validation of the cpzA3H–HIV-2 Vif interface and evolutionary conservation of interface residues. **a** Degradation assays of FLAG-tagged cpzA3H in the presence of HIV-2 Vif (H2Vif) alanine mutants at interface residues (R18A, G80A, F81A, L82A, H44A, H45A, W49A, W51A, W73A, and N74A). HEK293T cells were transfected with the indicated constructs, and cell lysates were probed for cpzA3H, H2Vif, and β-tubulin (β-tub) as a loading control. Wild-type (WT) and empty vector (−) serve as positive and negative controls, respectively. The results are representative from three independent experiments. **b** Degradation assays of FLAG-tagged cpzA3H alanine mutants at Vif-interacting residues spanning sub-interfaces 1, 2, and 3 (T35A, Q37A, C73A, Q75A, N103A, R105A, P132A, E134A; top panel; and R124A, C127A, E178A, K181A, Q182A, P139A, E140A; bottom panel), performed as described in (**a**). The results are representative of three independent experiments. **c, d** Sequence conservation of cpzA3H Vif-interacting residues across (**c**) catarrhines and (**d**) New World monkeys. Non-redundant, full-length A3H sequences were retrieved from the NCBI database, aligned using Clustal Omega^73^, and visualized with Logomaker^74^. The x-axis indicates cpzA3H residue positions comprising the Vif-interaction surface, and the y-axis shows information content in bits. Letter height reflects the degree of conservation, and letter color denotes amino acid physicochemical properties. Source data are provided as a Source Data file.

To validate the structural contacts, we performed Vif-induced degradation assays to assess how substitutions of specific residues involved in forming the HIV-2 Vif and cpzA3H interfaces affect Vif's ability to induce cpzA3H degradation. Mutational analysis of Vif identified W49 and W51 as critical determinants of cpzA3H degradation, forming a prominent tryptophan cluster (Fig. 3a). This result is consistent with biochemical findings by Smith et al., who identified these residues as critical determinants of HIV-2 Vif-induced degradation of human A3H haplotype II^46^. In contrast, Vif residues at the other two interfaces appear to be comparatively less critical for cpzA3H degradation, as individual alanine substitutions produced only small or negligible effects (Fig. 3a). This behavior is consistent with prior observations that functionally important interfaces can be distributed across multiple residues; for example, single substitutions within the HIV-1 Vif YRHHY motif do not significantly alter activity, whereas combined mutation of this motif (YRHHY to AAAAA) is required to disrupt APOBEC3G binding and degradation^47^. Because approximately twice as many cpzA3H residues as Vif residues participate in these intermolecular interactions, individual alanine substitutions in cpzA3H had minimal impact on its degradation (Fig. 3b). Accordingly, it is likely that simultaneous mutations of multiple residues in Vif, as well as in cpzA3H, would be required to achieve a more pronounced reduction in Vif-induced degradation.

We also performed conservation analysis to further highlight the potential functional relevance of interacting residues. Nearly all residues at the Vif–A3H interface are conserved between human A3H haplotype II and cpzA3H, with the exception of a conservative D/E substitution at position 178, supporting the relevance of the observed binding mode (Supplementary Fig. 6a and b). Consistent with the high conservation of interface residues, HIV-2 Vif efficiently degrades human A3H haplotype II in a CBFβ- and CUL5-dependent manner, and W49A/W51A substitutions markedly attenuate this degradation (Supplementary Fig. 6c), directly validating the translational relevance of our structural model. Sequence conservation of Vif interacting residues across catarrhines A3Hs reveals that residues T35, P36, Q37, C73, L104, P132, C127 and S183 are highly conserved or invariant, with R105 and K181 also strongly preserved as basic residues and E178 maintained as an acidic position (Fig. 3c). In contrast, Q75, N103, and E134 display moderate variability while largely retaining their physicochemical properties, whereas Q182 is more permissive. Notably, R124 is not broadly conserved as arginine, with most homologs harboring a leucine at the equivalent position, indicating lineage-specific variation at this site. Because HIV-2 does not infect New World monkeys, we compared Vif-binding residues of cpzA3H with those of New World monkey A3Hs, revealing greater sequence variability than in Old World monkey orthologues (Fig. 3d). P36, C73, N103, L104, R105, P132 and E134, E178 are invariant, whereas T35 and Q37 are strongly conserved. In contrast, Q75, R124 and C127 are not conserved in New World monkeys, consistent with lineage-specific divergence. Conservation further declines at the C terminus (K181, Q182, S183), where gaps and sequence heterogeneity indicate a species-specific region. Collectively, these results support the functional relevance of the residues involved in mediating contacts between HIV-2 Vif and cpzA3H.

### Comparative analysis of HIV-1 and HIV-2 Vif binding to cpzA3H

**Fig. 4:**
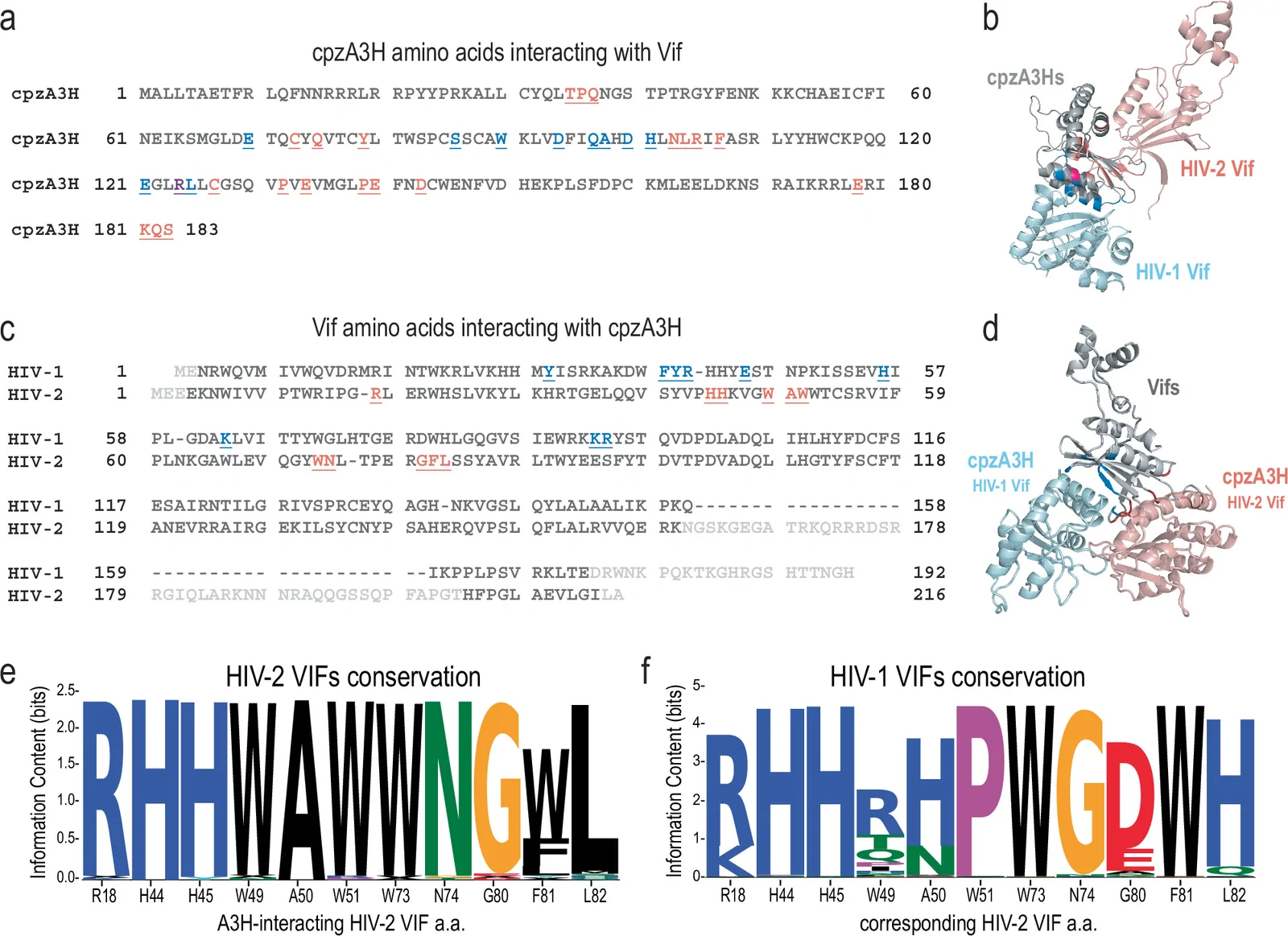
Comparative analysis of cpzA3H and Vif interface residues between HIV-1 and HIV-2. **a** Amino acid sequence of cpzA3H highlighting residues that interact with HIV-1 Vif (navy), HIV-2 Vif (salmon) and both HIV-1 and -2 (purple). **b** Superposition of cpzA3H (PDB: 5Z98 and this study) colored according to residues contributing to the interface with HIV-1 Vif (pale), HIV-2 Vif (salmon), or both (magenta). The structural model illustrates the divergence of the two interaction footprints. **c** Sequence alignment of HIV-1 and HIV-2 Vif highlighting cpzA3H-interacting residues (HIV-1, navy; HIV-2, salmon). **d** Superposition of HIV-1 Vif (PDB: 9E93) and HIV-2 Vif (this study), shown as a cartoon. Regions corresponding to the interface with cpzA3H are colored for HIV-1 Vif (blue), HIV-2 Vif (salmon), revealing divergent Vif surfaces engaged by cpzA3H. **e**, **f** Sequence conservation of cpzA3H-interacting residues across (**e**) HIV-2 Vif and (**f**) HIV-1 Vif sequences. Non-redundant, full-length Vif sequences were retrieved from the NCBI database, aligned using Clustal Omega^73^, and visualized with Logomaker^74^. The x-axis indicates HIV-2 Vif residue positions at the cpzA3H interaction surface, numbered according to HIV-2 Vif, and the y-axis shows information content in bits. Letter height reflects the degree of conservation across sequences, and letter color denotes amino acid physicochemical properties.

To understand how HIV-1 and HIV-2 counteract the same host restriction factor, we compared the binding interfaces of HIV-1 Vif (PDB: 9E93)^35^ and HIV-2 Vif (this study) on both the Vif and A3H surfaces. Structural superposition of the two complexes reveals that, despite conservation of the overall Vif fold, the residues contacting cpzA3H are largely non-overlapping between HIV-1 and HIV-2 (Fig. 4a), indicating that the two viruses engage distinct binding epitopes on cpzA3H.

The buried surface area between HIV-1 Vif and A3H is approximately 700 Å², compared to 845 Å² for HIV-2. Viewed from the cpzA3H molecule, the binding footprints of HIV-1 Vif (PDB:9E93, blue) and HIV-2 Vif (this study, salmon) occupy predominantly distinct regions (Fig. 4b). HIV-2 Vif engages a larger and more contiguous surface patch (28 residues are buried at the interface, 19 in direct contact) spanning the β-sheet platform and adjacent helical regions of cpzA3H. In contrast, HIV-1 Vif contacts a smaller region (19 residues are buried at the interface, 10 in direct contact), primarily localized to the α3 and α4 helices (Fig. 4c and d). Only a small region containing residues in the loop between β2 and β3 is shared between the two interfaces (Fig. 4d). Within this shared region, HIV-1 Vif residue E45 forms hydrogen bonds with cpzA3H residues S86 and W90, whereas HIV-2 Vif residues H45 and W49 mediate hydrophobic and van der Waals interactions with cpzA3H residues Y79, P139, and Y35, P36, Q37, C73, Q75, respectively.

A contact heatmap (Supplementary Fig. 7), derived from structural analysis by extracting residues likely involved in intermolecular contacts (<5.0 Å) in both complexes, further delineates the distinct sets of A3H residues engaged by each Vif protein, along with the corresponding Vif residues mediating these interactions. Several A3H residues are positioned at both Vif interfaces; however, only R124 contacts both Vif proteins, albeit via engaging distinct residues, *i.e*. F39 in HIV-1 Vif and F81 in HIV-2 Vif. From the Vif perspective, sequentially aligned residues are displayed along the x-axis, and only H42/H44 and T47/W49 (HIV-1/HIV-2) are suggested to form contacts (<5.0 Å) in both complexes, via different A3H residues (Supplementary Fig. 7). The complete separation of yellow dots (cpzA3H–HIV-1 Vif interactions) and blue dots (cpzA3H–HIV-2 Vif interactions) indicates that the intermolecular contacts are complex-specific, underscoring the distinct binding modes employed by HIV-1 and HIV-2 (Supplementary Fig. 7).

Sequence conservation analysis indicates that cpzA3H-interacting residues in HIV-2 Vif are under strong evolutionary constraint (Fig. 4e). The aromatic cluster W49, W51 and W73 is highly conserved, remaining almost invariant across sequences, consistent with a central role in stabilizing a hydrophobic interface or mediating protein–protein interactions. L82 is likewise strongly conserved as a hydrophobic residue, supporting a preserved nonpolar core. In contrast, F81 shows notable variability, accommodating predominantly aromatic or hydrophobic substitutions (W ~ 70%, F ~ 21%, L ~ 7%), indicating tolerance within a hydrophobic context. A50 and G80 are highly conserved while retaining small side chains, consistent with roles in structural packing or local flexibility. The polar residues N74, H44 and H45 are also strongly conserved, preserving hydrogen-bonding. Finally, R18 is nearly invariant as a basic residue, with only rare conservative substitutions, supporting a conserved electrostatic function. Comparison with HIV-1 Vif sequences shows that only a subset of positions are conserved (i.e. R18, H44, H45 and W73; Fig. 4f), indicating strong residue-level constraint. In contrast, HIV-2 Vif W51 is almost exclusively substituted by proline in HIV-1 Vifs, reflecting a lineage-specific replacement. The remaining positions (W49, A50, N74, G80, F81 and L82) are not conserved (Fig. 4f).

Taken together, direct comparison between HIV-1 and HIV-2 Vif proteins engage cpzA3H indicates highly divergent modes of binding, whereby distinct sets of residues within a given virus are evolutionarily constrained. This may reflect signatures of adaptation to their original primate hosts that are retained through host-pathogen evolution.

### A distinct CBFβ–A3H interface in the HIV-2 complex

**Fig. 5:**
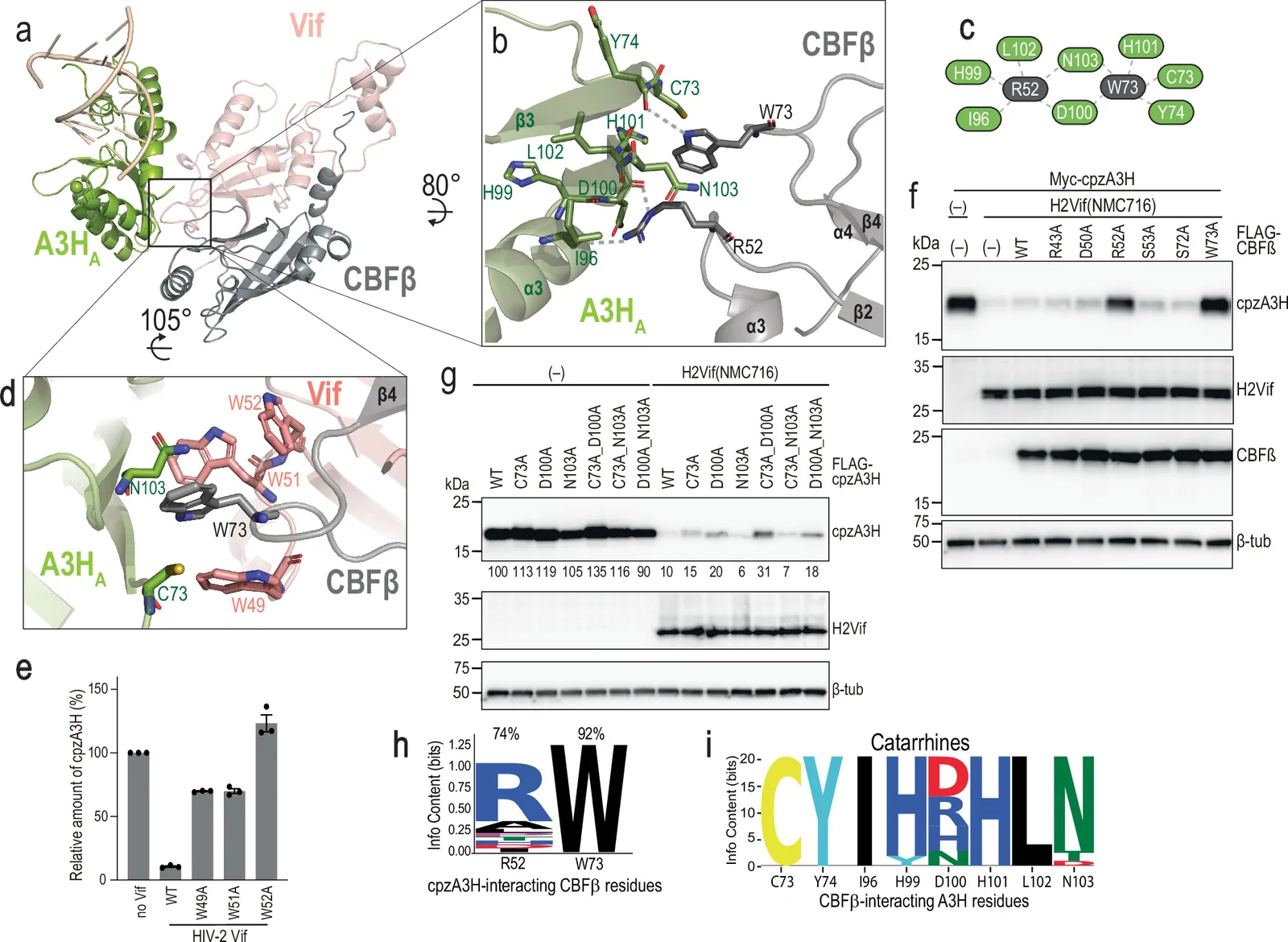
Molecular details of the CBFβ–cpzA3H interface and mutational validation. **a** Atomic model of the CBFβ (dark gray)–A3H_A_ (green) interaction shown in cartoon representation. The remaining components of the complex (A3H_B_, dsRNA, Vif, and EloBC) are shown as a semi-transparent surface for context. The boxed region indicates the CBFβ–A3H_A_ interface, shown in detail in (b), with the view rotated 80° relative to the main panel. **b** Close-up view of the CBFβ–cpzA3H interface, highlighting key interacting residues; Vif is omitted for clarity. CBFβ residues R52 and W73 engage cpzA3H residues on β3 (C73, Y74), α3 (I96, H99, D100), and β4 (H101, L102, N103). R52 forms multiple potential hydrogen bonds with backbone carbonyl groups of cpzA3H, while W73 contributes additional contacts through van der Waals interactions. Potential hydrogen bonds are indicated by dashed lines. **c** Schematic representation of the amino acid contacts at the CBFβ–cpzA3H interface. CBFβ residues are shown in dark gray and cpzA3H residues in green. **d** Close-up view of the CBFβ–cpzA3H–Vif interface highlighting key residues at the tripartite interaction surface. CBFβ W73 engages Vif residues W49, W51, and W52 to form a tryptophan cluster, which establishes hydrophobic and van der Waals interactions with cpzA3H residues C73 and N103. **e** HiBiT assay showing the effects of alanine substitutions of Vif tryptophan residues at the CBFβ–cpzA3H–Vif tripartite interface. Degradation activity is expressed as the percentage reduction in cpzA3H levels relative to the no-Vif control, normalized to wild-type HIV-2 Vif (WT). Data represent mean ± SEM from three independent experiments, with individual data points overlaid on the corresponding bars. **f** Effect of CBFβ alanine substitutions at the CBFβ–cpzA3H interface on cpzA3H degradation. Residues at and adjacent to the interface were individually substituted with alanine, and the stability of Myc-tagged cpzA3H was assessed by Western blotting using anti-Myc antibody 40 h post-transfection. Intracellular levels of H2Vif (NMC716) and FLAG-CBFβ were monitored using anti-His and anti-FLAG monoclonal antibodies, respectively. β-tubulin (β-tub) served as a loading control. Lanes 1 and 2 represent cpzA3H stability in the absence of both FLAG-CBFβ and H2Vif expression vectors, and in the absence of FLAG-CBFβ alone, respectively. The degradation of cpzA3H observed in lane 2 indicates that endogenous CBFβ is sufficient to support HIV-2 Vif–induced degradation. Wild-type FLAG-CBFβ (WT, lane 3) serves as a positive control for degradation. Results are representative of three independent experiments. **g** Effect of cpzA3H alanine substitutions at the CBFβ–cpzA3H interface on cpzA3H degradation. Individual cpzA3H interface residues were substituted with alanine, and the stability of FLAG-tagged cpzA3H was assessed by Western blotting using anti-FLAG antibody 40 h post-transfection. H2Vif(NMC716) levels were monitored using anti-HIS monoclonal antibody. β-tub served as a loading control. cpzA3H stability in the absence of H2Vif (−) serves as a negative control for degradation. Results are representative of three independent experiments. cpzA3H and β-tub band intensities were quantified using an ImageQuant LAS 4000 (Cytiva). cpzA3H levels were normalized to β-tub and expressed as a percentage relative to wild-type cpzA3H in the absence of HIV-2 Vif (NMC716, set to 100%). Normalized values are shown below each lane. **h**, **i** Sequence conservation of residues at the CBFβ–cpzA3H interface across (**h**) CBFβ from mammals and (**i**) A3H from catarrhines. The percentage conservation of key interface residues is indicated above the respective positions in (**f**). Non-redundant, full-length sequences were retrieved from the NCBI database, aligned using Clustal Omega^73^, and visualized with Logomaker^74^. The y-axis shows information content in bits, and letter height reflects the degree of conservation. Source data are provided as a Source Data file.

A distinctive feature of the HIV-2 Vif–cpzA3H structure is the presence of a direct interface between CBFβ and the proximal protomer of cpzA3H (cpzA3H_A_), which is absent in the HIV-1 Vif complex^35^ (PDB: 9E93). CBFβ contacts cpzA3H_A_ through a surface (buried surface area approximately 350 Å²) involving cpzA3H_A_ β-strands β2 and β4 and α-helix α4, with key contacts mediated by cpzA3H_A_ residues C73, Y74, I96, H99, D100, H101, L102, and N103, and by residues R52 and W73 on CBFβ (Fig. 5a, b). The interaction is stabilized by a network of hydrogen bonds and van der Waals contacts, as illustrated in the contact diagram (Fig. 5c).

The CBFβ–cpzA3H interface is mediated by a limited number of CBFβ residues that engage an extended surface on cpzA3H through distinct interaction modes (Fig. 5b, c). In particular, R52 forms multiple hydrogen bonds with backbone carbonyl groups of cpzA3H residues I96, H99, D100, and L102 (distances 3.0–3.3 Å), consistent with multidentate coordination by the guanidinium group. These interactions anchor R52 of CBFβ to a continuous segment of the cpzA3H backbone, suggesting a central role in stabilizing the interface through backbone-mediated recognition (Fig. 5b, c). An additional contact between R52 of CBFβ and the side chain of cpzA3H N103 (3.5 Å) is consistent with a weaker polar interaction. In contrast, W73 of CBFβ contributes through a combination of polar and hydrophobic contacts: its indole nitrogen is positioned to form a hydrogen bond with the backbone carbonyl of Y74 on cpzA3H_A_ (3.4 Å), while the aromatic ring engages surrounding residues, including H101, D100, C73, and N103, primarily through van der Waals interactions (3.1–3.7 Å) (Fig. 5b, c). Together, these observations indicate that only two CBFβ residues coordinate an extended interaction surface on cpzA3H_A_, with R52 providing a central anchoring function and W73 contributing additional stabilization through packing and a single polar contact.

The structure further reveals a cluster of tryptophan residues at the Vif–CBFβ–cpzA3H interface. Vif W49 and W51, key A3H binding residues, together with adjacent W52, flank CBFβ W73 to form a tightly packed assembly of four tryptophan residues (Fig. 5d). W52 does not directly contact cpzA3H but supports the cluster architecture. Although the aromatic side chains are not arranged in parallel, they converge toward the β sheet surface of cpzA3H and collectively engage residues N103 and C73 through hydrophobic and van der Waals interactions. Together, this arrangement forms a hydrophobic platform that bridges Vif and CBFβ on the cpzA3H surface. HiBiT assays show that alanine substitution of these Vif tryptophan residues markedly reduces cpzA3H degradation (Fig. 5e), and the tight local complementarity between this cluster and the cpzA3H β sheet suggests that this tryptophan assembly functions as a structural keystone of the interface. To further assess the biological relevance of these interactions, we performed mutational analyses targeting CBFβ and cpzA3H residues at the interface. Alanine substitution of CBFβ residues R52 and W73 rescued cpzA3H from Vif-induced degradation (Fig. 5f), underscoring the importance of the tryptophan cluster. The degradation of cpzA3H observed in lane 2 (Fig. 5f) indicates that endogenous CBFβ is sufficient to support HIV-2 Vif–induced degradation. Notably, neither the R52A nor W73A substitution affected degradation of APOBEC3G (A3G) or APOBEC3F (A3F) induced by HIV-2 Vif, nor did they alter cpzA3H degradation mediated by HIV-1 LAI Vif (Supplementary Fig. 8). Furthermore, CBFβ R52A and W73A substitutions impaired HIV-2 ROD10-mediated degradation of cpzA3H (Supplementary Fig. 8d), and W73A — but not R52A — rescued cpzA3H from SIVmac-induced degradation (Supplementary Fig. 8e), indicating that these interactions are partly conserved across HIV-2 and SIVmac lineages. In contrast, single-residue substitutions in cpzA3H did not produce pronounced rescue effects (Fig. 5g). Only the combined C73A/D100A substitutions conferred significant protection from degradation, consistent with structural data indicating that CBFβ engages an extended interaction surface on cpzA3H, predominantly through backbone-mediated and multivalent contacts.

Interestingly, CBFβ residues R52 and W73 are highly conserved across mammalian orthologues (Fig. 5h). R52 is retained as a positively charged residue (arginine or lysine), consistent with a conserved electrostatic function, whereas W73 is invariant, underscoring an essential structural role. This pattern reflects strong evolutionary constraint and functional importance. Conversely, CBFβ-interacting residues in A3H are also highly conserved across catarrhines (Fig. 5i). In particular, C73 and Y74 are invariant, whereas I96 and L102 are strongly conserved as hydrophobic residues. The H99–H101 segment is largely preserved, while D100 and N103 display limited but notable variation (D/H/R/N and N/T/D, respectively). Together, these patterns indicate a co-evolved and structurally conserved protein–protein interface that maintains key interaction features.

### Identification of ubiquitination sites on cpzA3H

**Fig. 6:**
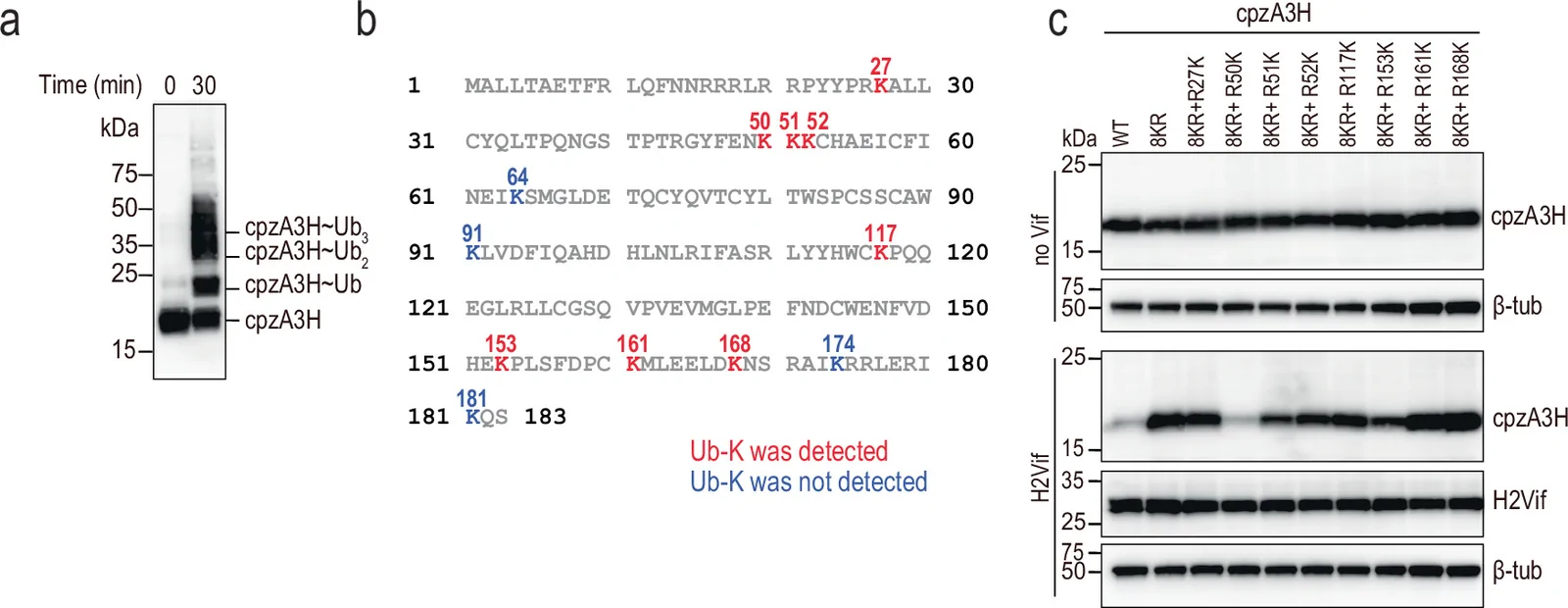
K50 is the primary ubiquitination site of cpzA3H targeted by HIV-2 Vif. **a** In vitro ubiquitination of cpzA3H by HIV-2 Vif (H2Vif). Reactions were incubated for 0 or 30 minutes and resolved by SDS-PAGE. Ubiquitinated cpzA3H species (cpzA3H~Ub, cpzA3H~Ub₂, and cpzA3H~Ub₃) were detected by Western blotting using an anti-A3H rabbit polyclonal antibody. The results are representative of three independent experiments. **b** cpzA3H amino acid sequence annotated with lysine residues identified by mass spectrometry as ubiquitination sites detected in the presence of HIV-2 Vif (red) or not detected as ubiquitinated (blue). Residue numbers are indicated above the respective lysines. **c** Identification of K50 as the critical lysine residue required for HIV-2 Vif–induced degradation of cpzA3H. A cpzA3H variant in which all eight lysines were mutated to arginine (8KR: K27R, K50R, K51R, K52R, K117R, K153R, K161R, and K168R) is resistant to HIV-2 Vif–induced degradation. Individual lysine residues were restored in the 8KR background to generate a series of single-lysine cpzA3H variants. Restoration of K50 alone (8KR + R50K) fully rescued HIV-2 Vif–induced degradation to a level comparable to wild-type cpzA3H. Cells were cultured in the absence (top panels) or presence (bottom panels) of HIV-2 Vif. All proteins were expressed without epitope tags and detected using anti-A3H rabbit polyclonal antibodies. β-tubulin (β-tub) served as a loading control. The results are representative of three independent experiments. Source data are provided as a Source Data file.

Identification of ubiquitination sites can provide mechanistic insight into how HIV-2 Vif targets cpzA3H for degradation. To this end, we reconstituted an in vitro ubiquitination system for cpzA3H using HIV-2 Vif (NMC716) in complex with the CUL5 E3 ligase machinery. All components of the assay (cpzA3H, the V2CBC complex, the CUL5–RBX2 complex, ARIH2, and UbcH7) were individually purified^35^. ARIH2 was charged with ubiquitin by UBE1 and UbcH7 to initiate cpzA3H monoubiquitination, followed by ubiquitin chain elongation, as previously described for HIV-1 Vif–mediated ubiquitination^35^. Ubiquitination of cpzA3H was confirmed by immunoblotting with an anti-A3H antibody, revealing a prominent ubiquitinated species (Fig. 6a). The reaction mixture was subsequently subjected to mass spectrometry (MS) analysis (see **Methods** for details). Although cpzA3H contains 12 lysine residues, we observed ubiquitination on only a subset of those, with K50, K51, and K52 being the most frequently ubiquitinated sites, and K27, K117, K153, K161, and K168 showing lower levels of ubiquitination (Fig. 6b).

To assess the functional relevance of these sites, we generated a cpzA3H mutant in which eight lysines identified by mass spectrometry were substituted with arginine (K27R, K50R, K51R, K52R, K117R, K153R, K161R, and K168R; hereafter referred to as cpzA3H-8KR). In HEK293T cells, this mutant exhibited substantial resistance to Vif-induced degradation (Fig. 6c**, lane 2**). Restoration of K50, but not K51 or K52, in the cpzA3H-8KR background reinstated Vif sensitivity, resulting in degradation levels comparable to wild-type (Fig. 6c**lane 4**), establishing K50 as the primary ubiquitination site for HIV-2 Vif-induced degradation. Together, these data demonstrate that K50 is the principal ubiquitination site for HIV-2 Vif-induced cpzA3H degradation, consistent with previous findings that K50 and K51 are the preferred ubiquitination sites for HIV-1 Vif-induced A3H degradation^34,35^.

### Ubiquitination mechanism of cpzA3H by V2CBC-CUL5 E3 ligase

**Fig. 7:**
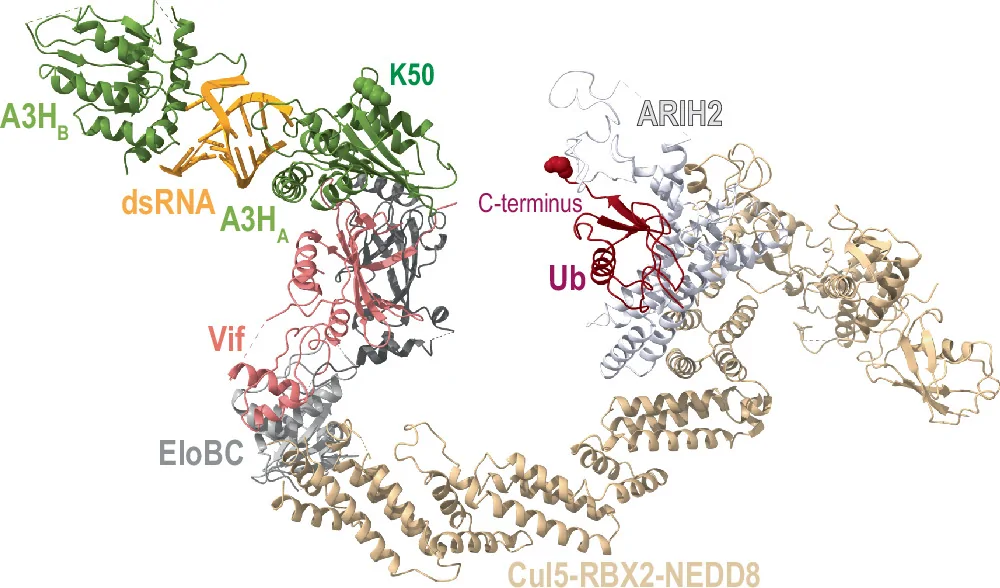
Mechanistic model of cpzA3H ubiquitination by the HIV-2 Vif–CUL5 E3 ligase complex. Composite model of the complete ubiquitination machinery assembled on cpzA3H, built by integrating the high-resolution cryo-EM structure of the Vif–cpzA3H–CBFβ–EloBC core complex determined in this study (3.08 Å) with components modeled by rigid-body fitting of published structures (PDB: 7B5M, 7ONI, and 8FVI) into lower-resolution density. Components are colored as follows: A3HA and A3HB, green; dsRNA, orange; HIV-2 Vif, salmon; CBFβ, dark gray; EloBC, light gray; Cul5–RBX2–NEDD8, beige; ARIH2, lavender; ubiquitin (Ub), maroon. The C-terminal glycine of ubiquitin (Gly76) and the target lysine of cpzA3H (K50) are shown as spheres. The model reveals how the Vif–CBFβ–EloBC adaptor module bridges cpzA3H substrate recruitment and the elongated Cul5 scaffold, positioning the ubiquitin-charged ARIH2 E2-like enzyme in Transition State 2 for mono-ubiquitination of A3H_A_. Note that the positioning of the CUL5–RBX2–ARIH2 module and the ubiquitination geometry of K50 represent a structurally informed model consistent with the experimental density, rather than features directly resolved at high resolution.

To further contextualize the A3H ubiquitination mechanism, we collected cryo-EM data for the reconstituted CUL5–RBX2–cpzA3H–V2CBC complex, assembled by combining equimolar amounts of CUL5–RBX2, the cpzA3H–dsRNA dimer, and V2CBC, followed by isolation of the intact assembly via size-exclusion chromatography (Supplementary Fig. 1i). Despite extensive optimization, preferred particle orientation limited map quality and precluded de novo model building of the full complex. To address this, we implemented a masking strategy (cryoMaskR^48^) for automated soft-mask optimization within standard CryoSPARC workflows, enabling improved reconstruction of a subregion encompassing cpzA3H–V2CBC and the N-terminal domain of CUL5. Rigid-body fitting of the cpzA3H–V2CBC structure (this study) together with the N-terminal CUL5–HIV-1 Vif–CBFβ atomic model (PDB: 8FVI) accounted for this density (Supplementary Figs. 9, 10). Additional density corresponding to the C-terminal Cul5–RBX2 module was consistent with canonical Cullin architecture and supported placement of elements from the Cul1–RBX1 complex (PDB: 7B5M). Integration of these components yielded a composite model of the fully assembled cpzA3H–V2CBC–CUL5 E3 ligase (Fig. 7). The cpzA3H–V2CBC-nCul5 module is directly resolved in our cryo-EM map, while the CUL5–RBX2 scaffold and ARIH2 ubiquitin transfer machinery were positioned by rigid-body fitting of existing structures into the lower-resolution peripheral density. ARIH2 (PDB: 7ONI) was superimposed onto the ubiquitin-primed ARIH1 structure from PDB: 7B5M (Transition State 2^49^; catalytic step of ubiquitin transfer), yielding a complete structural framework consistent with the experimental data and enabling interpretation of the spatial relationship between the ligase and putative ubiquitin-acceptor lysines on cpzA3H. Consistent with our biochemical data identifying K50 as the preferred ubiquitination site (Fig. 6c), the model positions this residue on the Vif-proximal cpzA3H protomer in close spatial proximity to the C-terminal glycine of the conjugated ubiquitin, providing a structural rationale for substrate-specific ubiquitin transfer. Since ARIH2 recruits E2 ubiquitin-conjugating enzymes prior to ubiquitin transfer to target proteins, we have generated another structural model (Supplementary Fig. 11), depicting ARIH2 bound to the E2 enzyme UBE2L3 (Transition State 1^49^). This structure represents the pre-catalytic E3-E2 complex that precedes ubiquitin transfer to A3H_A_.

## Discussion

The structure of the HIV-2 Vif–cpzA3H complex provides direct evidence that primate lentiviral antagonism of APOBEC3 proteins has evolved through divergent structural solutions for convergent functional tasks. Although HIV-1 and HIV-2 Vif share a conserved overall fold, they engage cpzA3H through largely non-overlapping interfaces with minimal shared contact residues, indicating that the Vif scaffold serves as a versatile platform for evolving distinct substrate-recognition surfaces. This interface plasticity is consistent with strong selective pressure imposed by rapidly evolving APOBEC3 proteins^50–52^, positioning A3H as a moving evolutionary target. The near-complete separation of binding footprints demonstrates that HIV-1 and HIV-2 have converged on mechanistically equivalent, yet structurally distinct, solutions to the same antiviral challenge^37,38^. In this context, conservation of residues at the HIV-2 Vif–cpzA3H interface highlights a functionally constrained interaction optimized for efficient antagonism in the relevant primate host. Sustained preservation of these key contacts underscores the requirement to maintain robust A3H counteraction for productive infection, whereas their divergence from HIV-1 Vif interaction sites illustrates how distinct lentiviral lineages independently remodel their interfaces to accommodate species-specific variation in A3H. Together, these findings support a model in which Vif evolves through targeted reconfiguration of its interaction surface, enabling host adaptation and cross-species transmission while preserving a conserved structural core. Within this framework, HIV-2 Vif appears, based on evolutionary inference, to retain ancestral features of SIVsmm Vif that are broadly compatible with primate APOBEC3 proteins, consistent with the ability of primary SIVsmm isolates to replicate in human peripheral blood mononuclear cells^53^. In contrast, HIV-1, having overcome the cross-species barrier through recombination of multiple SIV lineages (reviewed in ref. ^54^), appears to have undergone more extensive co-evolution with human APOBEC3 proteins to establish specialized interaction modes. Previous studies have reported elevated A3F/A3G-mediated editing signatures in HIV-2 genomes from patients compared with HIV-1^55^, suggesting less complete APOBEC3 antagonism by HIV-2 Vif. This observation raises the possibility that differential APOBEC3 restriction contributes to the reduced pathogenicity of HIV-2, although this remains to be directly tested. Although our structure is based on cpzA3H, 20 of 21 Vif-contacting residues are conserved in human A3H haplotype II. Consistently, HIV-2 Vif degrades human A3H haplotype II in a CBFβ- and CUL5-dependent manner, and W49A/W51A substitutions markedly attenuate this degradation (Supplementary Fig. 6c), validating the translational relevance of our model. Nonetheless, haplotype-specific differences in protein stability, subcellular localization, and RNA binding may modulate degradation efficiency, and future studies incorporating all human A3H haplotypes will be needed to fully define the basis of haplotype-dependent Vif susceptibility.

A key insight from this study is the expanded role of CBFβ in the HIV-2 complex. Beyond its established function in stabilizing Vif^28,29,56^, CBFβ directly engages cpzA3H, contributing to substrate recruitment. This interface, mediated by a limited number of residues spanning an extended A3H surface, supports a model in which CBFβ acts as a bivalent adaptor bridging Vif and substrate, thereby enhancing binding avidity and potentially orienting A3H for ubiquitination. While CBFβ has been shown to directly interact with A3F in HIV-1 Vif complex through distinct electrostatic interfaces (CBFβ R35/R43 with A3F E324, and CBFβ E54 with A3F R293) that are critical for A3F degradation but dispensable for A3G targeting^30^, the absence of analogous A3H-CBFβ interactions in HIV-1 Vif complexes^34,35^ suggests a lineage-specific adaptation and indicates that CBFβ can play a more active, context-dependent role in substrate positioning than previously appreciated. These findings expand our understanding of how different HIV lineages have evolved distinct CBFβ-mediated substrate recognition mechanisms to counteract the diverse A3 repertoire. While this manuscript was under preparation, an independent study reported the cryo-EM structure of HIV-2 Vif bound to human A3H and CBFβ, similarly revealing a direct CBFβ–A3H interface and a mode of A3H recognition distinct from that of HIV-1 Vif complexes^57^. The convergence of these independent findings across A3H orthologues indicates that CBFβ-mediated substrate engagement is a conserved feature of the HIV-2 Vif–CBFβ–A3H assembly, rather than a species-specific property of the cpzA3H complex described here.

Despite these differences in binding mode, HIV-1 and HIV-2 Vif converge on a shared ubiquitination outcome, preferentially targeting K50 of A3H. This convergence suggests that substrate positioning, rather than precise interface composition, is the primary determinant of ubiquitination efficiency. Distinct Vif–A3H interfaces appear to dock A3H in a geometry that presents K50 to the E2–E3 catalytic center, implying geometric constraints imposed by the Cullin-RING ligase architecture^58–63^. Consistent with this model, restoration of K50 alone is sufficient to recover Vif-mediated degradation (this study). It should be noted that the ubiquitination geometry of K50 is inferred from rigid-body docking of the CUL5–RBX2–ARIH2 assembly into lower-resolution density and represents a structurally informed model rather than a directly resolved feature. Higher-resolution reconstruction of the complete CUL5 assembly will be required to confirm the precise geometry of ubiquitin transfer. Together, these findings support a framework in which Vif–substrate interfaces are evolutionarily flexible but constrained at the level of ubiquitin transfer geometry.

An additional distinction revealed by this structure is the apparent RNA independence of HIV-2 Vif-mediated A3H recognition. In contrast to HIV-1 systems, where RNA can act as a structural bridge in Vif–APOBEC3 interactions, particularly for A3G^31–33^, the HIV-2 Vif–A3H interface is composed entirely of direct protein–protein contacts. Although A3H itself forms a dsRNA-mediated dimer required for stability and function^19–21^, RNA does not participate in Vif binding. This suggests that HIV-2 Vif has evolved to bypass RNA as an architectural cofactor, instead relying on direct surface complementarity. More broadly, these findings highlight two mechanistic paradigms in A3 antagonism: RNA-dependent interfaces that incorporate nucleic acids as structural elements, and RNA-independent interfaces that rely solely on protein contacts.

In conclusion, our findings establish a structural framework in which diverse viral interfaces converge on a common functional outcome, highlighting the interplay between evolutionary diversification, substrate-specific recognition, and mechanistic constraints in ubiquitin-mediated antagonism of host restriction factors.

## Methods

### cpzA3H protein expression and purification

GST-cpzA3H was purified as previously described, without modifications^35^. Briefly, pET-41 GST-cpzA3H was transformed into Rosetta 2(DE3)pLysS competent cells (Merck Millipore) and grown in Luria-Bertani (LB) medium supplemented with kanamycin (50 µg/mL) and chloramphenicol (34 µg/mL) at 37 °C until OD600 ~ 0.6. Cells were placed on ice for 15 min, and protein expression was induced with 10 mg/ml lactose monohydrate in the presence of 1 µM ZnSO4 for 18 h at 20 °C. Cells were harvested by centrifugation (7000 × *g*, 4 °C, 15 min) and resuspended in lysis buffer [50 mM Tris-HCl, pH 7.4, 1 M NaCl, 10%(v/v) glycerol, 5 mM 2-mercaptoethanol (2-ME), 1%(v/v) Triton-X100, 500 µg/mL RNase A (Qiagen)]. The cells were lysed by sonication, centrifuged at 28,620 × *g* for 30 min. The supernatant was filtered through a 0.8-µm pore-size membrane and applied to a glutathione Sepharose 4 Fast Flow (FF) column (Cytiva), pre-equilibrated with the lysis buffer. The GST-tagged protein was eluted with elution buffer A containing 50 mM Tris-HCl, pH 7.4, 500 mM NaCl, 2%(v/v) glycerol, 1 mM 2-ME, 40 mM reduced L-glutathione. After treatment with thrombin (33.3 U/mL) (Cytiva) and RNase A (1.67 mg/mL) at 20 °C for 16 h, the cpzA3H was further purified using size exclusion chromatography. HiLoad 26/600 Superdex-75 column (Cytiva) was pre-equilibrated with gel filtration buffer A containing 25 mM HEPES, pH 7.4, 500 mM NaCl, 0.25%(v/v) glycerol, and 1 mM Tris(2-carboxyethyl)phosphine (TCEP). To remove the residual GST tag, the solution was loaded onto a Ni-Sepharose 6 Fast Flow (FF) (Cytiva) column in tandem with a glutathione Sepharose 4 FF column. The flow-through fraction containing the cpzA3H recombinant proteins was concentrated to 30 µM using an Amicon Ultra 15 mL 10 K (Merck Millipore) and stored in –80 °C.

### HIV-2 Vif/HIS6-CBFß/EloB/EloC protein expression and purification

The codon-optimized Vif ORF fragment derived from pHIV-2 NMC716 proviral clone (GenBank# LC927253) was synthesized commercially (Fasmac). A Vif ORF fragment derived from the HIV-2 NMC716 was amplified by PCR and cloned into pET-Duet. Purification of HIV-2 Vif/HIS6-CBFß/EloB/EloC complex (V2CBC) was performed as follows: N-terminal hexa-HIS-tagged CBFβ/HIV-2 Vif (pET-Duet) and codon-optimized EloC/EloB (pRSF-Duet) were transformed to One Shot BL21 Star (DE3) Chemically Competent E. coli cells (Thermo Fisher Scientific) and grown in LB medium containing ampicillin (75 µg/mL) and kanamycin (50 µg/mL) at 37 °C until OD600 of ~0.6. After cooling on ice for 15 min, protein expression was induced with 0.5 mM Isopropyl β-D-1-thiogalactopyranoside (IPTG) in the presence of 10 µM ZnSO4 at 20 °C for 18 h. Cells were harvested by centrifugation at 7000 × *g* at 4 °C for 15 min and resuspended in lysis buffer B containing 50 mM Tris-HCl, pH 8.0, 500 mM NaCl, and 5 mM 2-ME, 1% TritonX-100. Cells were sonicated, spun down at 23,000 × *g* for 30 min at 4 °C, and filtered through a 0.8-µm pore-size membrane. The cleared soluble fraction was applied to a Ni-Sepharose 6 FF (Cytiva). The column was washed with 40 column volumes of wash buffer [50 mM Tris-HCl, pH 8.0, 500 mM NaCl, and 40 mM imidazole]. The V2CBC complex was eluted with elution buffer B: 50 mM Tris-HCl, pH 8.0, 500 mM NaCl, 300 mM imidazole, 5 mM 2-ME. The sample was diluted to the final NaCl concentration of 100 mM, applied to Q cation-exchange chromatography (Cytiva). The protein was eluted with a linear gradient (buffer A: 25 mM Tris HCl, pH 8.0, 200 mM NaCl, 5 mM 2-ME; buffer B: 25 mM Tris HCl, pH 8.0, 500 mM NaCl, 5 mM 2-ME). The V2CBC complex was further purified using a Hiload 26/600 Superdex 200 column equilibrated with gel filtration buffer A and concentrated to 30 µM using Amicon Ultra 15 mL 10 K (Merck Millipore), and stored in –80 °C.

### Cul5/RBX2 protein expression and purification

Purification of HIS6-tagged CUL5/RBX2 was performed as previously reported with slight modifications^35^. Plasmids HIS6-tagged CUL5/RBX2 (pRSF-Duet) and RBX2 (pET) were co-transformed into One Shot BL21 Star (DE3) Chemically Competent E. coli and grown in LB medium containing ampicillin (75 µg/mL) and kanamycin (50 µg/mL) at 37 °C till OD600 of ~0.6. After cooling on ice for 15 min, protein production was induced with 0.5 mM IPTG in the presence of 10 µM ZnSO4 at 16 °C for 18 h. Cells were harvested by centrifugation at 7000 × *g* at 4 °C for 15 min, resuspended in lysis buffer B, and sonicated. Lysate was cleared by centrifugation at 23,000 × *g* for 30 min at 4 °C, filtered through a 0.8-µm pore-size membrane, and applied to a Ni-Sepharose 6 FF. The column was washed with 40 column volumes of wash buffer, and the protein complex was eluted using elution buffer B. The heterocomplex was further purified using a Hiload 26/600 Superdex 200 column equilibrated with gel filtration buffer A, and concentrated to 30 µM by using Amicon Ultra 15 mL 30 K (Merck Millipore).

### ARIH2 protein expression and purification

For recombinant ARIH2 protein purification, the E. coli expression system was used as previously described with slight modifications^35^. pET41-GST-ARIH2 plasmid was transformed into One Shot BL21 Star (DE3) Chemically Competent E. coli. Cells were grown in LB medium containing kanamycin (50 µg/mL) at 37 °C to an OD600 of ~0.6. After cooling down on ice for 15 min, the protein production was induced with 0.5 mM IPTG in the presence of 10 µM ZnSO4 at 16 °C for 18 h. Cells were harvested by centrifugation at 7000 × *g* at 4 °C for 15 min, resuspended in lysis buffer B, and sonicated. Lysate was cleared by centrifugation at 23,000 × g for 30 min at 4 °C, filtered through a 0.8-µm pore-size membrane, and applied to a glutathione Sepharose 4 FF column for affinity purification. The GST-ARIH2 protein was eluted with elution buffer A and treated with thrombin (33.3 U/mL) (Cytiva) at 20 °C for 16 h. The protein was loaded onto a Hiload 26/600 Superdex-200 column (Cytiva) pre-equilibrated with gel filtration buffer B [25 mM Tris-HCl (pH 8.0), 500 mM NaCl, and 5 mM 2-ME]. To remove the residual GST tag, the protein solution was loaded onto a glutathione Sepharose 4 FF column. The fraction containing the ARIH2 recombinant proteins was concentrated to 40 µM using an Amicon Ultra 15 mL 30 K (Merck Millipore).

### UbcH7 (UBE2L3) protein expression and purification

His6-UbcH7 was purified as previously described^35^. Briefly, pET-His6-UbcH7 was transformed into One Shot BL21 Star (DE3) *E. coli* (Thermo Fisher Scientific), and cells were grown in LB medium with ampicillin (75 µg/mL) at 37 °C to OD600 = 0.6. Protein expression was induced with 0.5 mM IPTG for 18 h at 20 °C. Cells were harvested (7,000 × g, 4 °C, 15 min), resuspended in lysis buffer B, and lysed by sonication. The lysate was clarified (23,000 × g, 4 °C, 30 min), filtered (0.8 µm), and purified by Ni-Sepharose 6 FF (Cytiva) affinity chromatography, followed by size-exclusion chromatography (HiLoad 26/600 Superdex 75, Cytiva) in gel filtration buffer B. Purified protein was concentrated to 25 µM using a Centriprep 10 K concentrator (Merck Millipore), with purity assessed by SDS-PAGE at each step.

### N-terminal Cul5 (nCul5) protein expression and purification

nCul5 was purified as previously described^34^. Briefly, GST-nCUL5 (residues 12–320) was expressed in *E. coli* BL21(DE3) grown in LB medium with ampicillin (100 µg/mL) at 37 °C to OD600 = 0.6, and expression was induced with 0.5 mM IPTG for 18 h at 16 °C. Cells were harvested by centrifugation and stored at −80 °C. Pellets were resuspended in lysis buffer A and lysed by sonication, and the lysate was clarified by centrifugation (23,000 × g, 4 °C, 30 min). The supernatant was incubated with glutathione Sepharose resin (Cytiva) for 1 h at 4 °C, and the resin was washed extensively with lysis buffer A. The GST tag was cleaved on-column with PreScission protease overnight, and nCUL5 was eluted and further purified by size-exclusion chromatography (HiLoad 16/600 Superdex 75 pg, Cytiva) in gel filtration buffer B. Peak fractions were pooled and concentrated to 40 µM using an Amicon Ultra concentrator (Merck Millipore).

### Cryo-EM data acquisition

cpzA3H-V2CBC-nCul5 complex was formed by mixing the purified V2CBC complex and cpzA3H in 1:1 molar ratio. After incubating on ice for 15min, the protein sample was applied to the HiLoad 10/300 Superose6pg column (Cytiva) in a buffer containing 25mM Tris-HCl, pH 8.0, 350mM NaCl, 0.25%(v/v) glycerol, and 1mM TCEP. Each fraction of freshly purified cpzA3H-V2CBC complex was diluted to ABS280 = 0.4, and 3.5 μL aliquots were applied to Quantifoil Cu R0.6/1.0 holey carbon 200-mesh grids (Electron Microscopy Sciences), glow discharged for 15 sec, 25 mA. Grids were blotted and vitrified in liquid ethane using Vitrobot Mark IV (Thermo Fisher Scientific) at 6 °C with 100% humidity.

Cryo-EM data of the cpzA3H-VCBC-nCul5 complex were collected using a Talos Arctica G2 electron microscope (Thermo Fisher Scientific) equipped with a K3 direct electron detector and an energy filter, operated at 200 kV in electron-counting mode. Movies were collected at a nominal magnification of 100,000× in super resolution mode (0.405 Å/pixel). 40 frames per movie were acquired, with a total dose of 50 e − /Å2 and a total exposure time of approximately 2.5 s. A total of 19,563 movies were recorded by automated data acquisition with EPU (Thermo Fisher Scientific), with defocus values ranging from –2.2 to –0.8 μm.

### Cryo-EM data processing

The movies were imported into cryoSPARC 4.5.3 software package^64^ and subjected to patch motion correction, binning to physical pixel size, and CTF estimation. Movies with CTF resolution worse than 5 Å and relative ice thickness >1.1 were removed, leaving 17,969 movies for further processing. Reference-free blob particle picking was performed on denoised micrographs. A total of 7,413,018 particles were picked initially, extracted, and down sampled by a factor of 4. The iterative rounds of 2D and ab initio classifications were performed. 2,735,174 particles were re-extracted with full resolution. After heterogeneous refinement, re-balancing orientations, reference motion correction, and non-uniform refinement were performed, 294,824 particles were used to reconstruct a 3.08Å resolution map (Supplementary Fig. 2). To further improve the quality and interpretability of the calculated maps, we added a post-processing step with EMready2.041. All resolution evaluation was performed based on the gold-standard criterion of the FSC coefficient at 0.143^65^.

### Model building and refinement

For the cpzA3H-V2CBC complex, atomic models derived from the crystal structure of cpzA3H (PDB ID: 5Z98)^21^ and the V2CBC model generated by AlphaFold3^66^ were docked into the cryo-EM map using UCSF ChimeraX^67^. The model was refined with the phenix.real_space_refine module in Phenix, with secondary structure and geometry restraints^68^. The protein main chains and side chain rotameters were adjusted manually in COOT^69^ to match the calculated map. Both atomic models were subjected to iterative cycles of real-space refinement in Phenix and manual adjustments in COOT. The final model was validated by tools available in Phenix (Table 1) All structural figures were generated using UCSF ChimeraX^67^ or PyMOL^70^.

**Table 1:** Cryo-EM data collection, refinement and validation statistics

Data collection and processing	
Microscope	Talos Arctica
Detection camera	Gatan K3
Voltage (kV)	200
Magnification	100,000
Electron exposure (e − / Å^2^)	50
Defocus range (μm)	0.8 − 2.2
Pixel size (Å)	0.81
Symmetry imposed	C1
Initial particle images (no.)	7,413,018
Final particle images (no.)	294,824
Map resolution (Å)	3.08
FSC threshold	0.143
Map resolution range (Å)	2.6-4.2
**Model Building and Refinement**	
Initial model used (PDB code)	4N9F, 5Z98, AlphaFold3
Model resolution (Å)	3.14
FSC threshold	0.5
Model resolution range (Å)	2.54-6.2
Map sharpening B factor (Å)^a^	98.3
B factors (Å^2^)	
Protein	61.53
Nucleotide	41.99
Ligand	97.40
*R.m.s. deviations*	
Bond lengths (Å)	0.003
(outliers >4 sigma)	0
Bond angles (°)	0.561
(outliers >4 sigma)	5
*Validation*	
MolProbity score	1.58
Clashscore	11.58
Rotamer outliers (%)	0.0
CaBLAM outliers (%)	1.3
Cβ outliers (%)	0
*Ramachandran plot*	
Favored (%)	98.96
Allowed (%)	1.04
Outliers (%)	0.0
*Model composition*	
Nonhydrogen atoms	6,968
Protein residues	797
Nucleotide residues	19
Ligands	ZN: 3
*Real-space correlation*	
CCvolume	0.84
CCmask	0.83

cpzA3H–V2CBC (EMDB-75888), (PDB 11ON)

### Vif-mediated degradation assay

We used pTR600 FLAG-cpzA3H WT, myc-cpzA3H WT, and cpzA3H WT (no tag) as previously described^35^. We used pcDNA-HVif LAI (Myc-His), human A3H haplotype II WT, and FLAG-cpzA3H R124A plasmids as previously described^25^. We also used pcDNA FLAG-CBF-β WT plasmids as previously described^71^. For the pcDNA A3G WT (no tag) or pcDNA A3F WT (no tag) expression plasmid, the DNA fragment was amplified by PCR from pcDNA A3G-mycHIS or pcDNA A3F-mycHIS plasmid DNA^72^ using the PrimeSTAR Max DNA Polymerase (Takara Bio); the primer sets used are listed in Supplementary Table 1. The codon-optimized Vif ORF fragment derived from the HIV-2 ROD Vif (GenBank# M15390) or SIVmac (GenBank# AY588946.1) was synthesized commercially (Fasmac). The pHIV-2 NMC716 proviral clone was used as PCR templates for Vif ORF fragment derived from HIV-2 NMC716 Vif. For the pcDNA H2Vif(NMC716)-Myc-HIS, pcDNA H2Vif(ROD10)-Myc-HIS or pcDNA SIVmac Vif-Myc-HIS expression plasmid, we amplified the HIV-2 Vif DNA fragments by PCR using PrimeSTAR Max DNA Polymerase (Takara Bio); primer sequences are available in Supplementary Table 1. For each CBFß or cpzA3H mutant, substitution mutations were introduced by oligonucleotide-directed PCR using appropriate primer sets (Supplementary Table 1). For the pTR600 cpzA3H 8KR or revertant mutant plasmid, the oligonucleotide-directed PCR was used using appropriate primer sets (Supplementary Table 1) from the pTR600 cpzA3H 7KR or 7KR revertant mutant plasmid^35^. For analysis of the Vif-mediated cpzA3H, A3G, and A3F degradation using CBFß mutants, the cpzA3H expression plasmid (0.25 µg), Vif expression plasmid (0.5 µg), and CBFß expression plasmid (2.25 µg) were co-transfected into HEK293T cells in 12-well plates using FuGENE HD (Promega). For analysis of the Vif-mediated cpzA3H degradation using FLAG-cpzA3H and cpzA3H 8KR mutants, the cpzA3H expression plasmid (0.25 µg) and Vif expression plasmid (2.75 µg) were co-transfected into HEK293T cells in 12-well plates using FuGENE HD (Promega). At 40 h post-transfection, the cells were lysed using Laemmli buffer (Bio-Rad) in the presence of 2.5% (vol/vol) 2-ME. To analyze the protein expression levels, we fractionated HEK293T cell lysates by SDS-PAGE using e-PAGEL 12.5% gels (Atto Co.) and transferred them onto Immobilon-P membranes (Merck-Millipore). The membranes were first probed with the appropriate primary antibodies. The myc-cpzA3H was detected with an anti Myc-Tag (9B11) mouse monoclonal antibody (mAb) (1:2,000; Cell signaling; cat # 2276S). The cpzA3H (no tag) and cpzA3H 8KR were detected with anti-A3H rabbit polyclonal antibody (1:2,000; Novus Biologicals; cat #NBP1-91682). The A3F (no tag) was detected with anti-A3F rabbit polyclonal antibody (1:2,000) that was generated against the C-terminal 18 amino acids of human A3F in rabbit via the custom production services (Medical & Biological Laboratories Co.). The A3G (no tag) was detected with an anti-Human A3G (ApoC17) polyclonal antibody (1:2,000; AIDS Research and Reference Reagent Program; cat #ARP-10082)^3^. The FLAG-cpzA3H and FLAG-CBFß was detected with an anti-DYKDDDDK (FLAG)-tagged mouse monoclonal antibody (mAb) (1:2,000; Fujifilm Wako Pure Chemical Co.; cat #012-22384). The HIV-1 and HIV-2 Vif proteins were detected using a mouse anti-HIS monoclonal antibody (1:2,000; Medical & Biological Laboratories Co.; cat #D291-3), and β-tubulin, which was used as a loading control, was detected with a rabbit anti-β-tubulin polyclonal antibody (1:2000; Abcam; cat #ab6046). The immunoblotted membranes were subsequently incubated with horseradish peroxidase-conjugated secondary antibodies. A goat anti-mouse IgG antibody (1:20,000; Thermo Fisher Scientific) and a goat anti-rabbit IgG antibody (1:20,000; Thermo Fisher Scientific) were used for Myc, FLAG, HIS, A3G, A3F, and cpzA3H and β-tubulin, respectively. The proteins were visualized by enhanced chemiluminescence using SuperSignal West Dura (Thermo Fisher Scientific) and quantified using an ImageQuant LAS 4000 (Cytiva).

### HiBiT assay

For the HiBiT assay, pTR600 HiBiT-FLAG-cpzA3H was constructed to express cpzA3H carrying an N-terminal HiBiT-FLAG tag. Briefly, the pTR600 FLAG-cpzA3H WT plasmid was used to insert the 12-amino acid HiBiT tag-coding sequence, and the plasmid DNA fragment was amplified by high-fidelity inverse PCR using PrimeSTAR GXL DNA polymerase (Takara Bio). Primers used were (5´- ATGGTGAGCGGCTGGCGGCTGTTCAAGAAGATTAGCGGCGATTACAAGGATGACGACGATAAG and 5´- CAGCCGCCAGCCGCTCACCATGGCAAGCTTGGACGGTGACTGCAGAAAAGACC). The resultant fragment was self-joined with the NEBuilder HiFi DNA Assembly reagent (New England Biolabs) and transformed into the competent E. coli DH5α cells. pTR600 HiBiT-FLAG-cpzA3H was cloned and the sequences of both the cpzA3H and boundary regions were verified by DNA sequencing.

To quantify the intracellular HiBiT-FLAG-cpzA3H level in the presence of H2Vif, transfection for the HiBiT assay was performed in 96-well plates using HEK293T cells (1.5 × 10^4^ cells per well) that were seeded 24 h prior to transfection. The HiBiT-FLAG-cpzA3H expression plasmid (84 ng), the H2Vif-mycHIS expression plasmid (210 ng), and a firefly luciferase expression plasmid were co-transfected into the cells using FuGENE HD (0.63 µL per well) according to the manufacturer’s instructions. Transfections were performed in duplicate plates to quantify NanoLuc and firefly luciferase luminescence. At 40 h post-transfection, the transfected cells were subjected to a HiBiT assay using the Nano-Glo HiBiT Lytic Detection System (Promega) according to the manufacturer’s instructions. First, the cells in one plate were assayed for NanoLuc luminescence by adding the Nano-Glo HiBiT Lytic substrate and LgBiT protein in Lytic Buffer (75 µL, a volume equal to the cell culture medium). The cell lysate was incubated at room temperature for 10 min, and 50 µL of the lysate mixture was transferred to a white 96-well plate. The NanoLuc luminescence was measured using an ARVO MX luminescence counter (PerkinElmer). The firefly luciferase activity in the second plate was assayed using the Steady-Glo Luciferase Assay System (Promega). The transfected cells were lysed with the Steady-Glo Luciferase Assay Buffer containing substrate, and the firefly luminescence was measured in the counter. Firefly luminescence in each well, relative to wells lacking HiBiT-FLAG-cpzA3H and H2Vif-mycHIS expression, was calculated as relative transfection efficiency (%). Each of the NanoLuc luminescence values was subsequently normalized by dividing by the relative transfection efficiency in the corresponding well. The steady-state levels of cpzA3H with H2Vif relative to levels without H2Vif were obtained as the “relative amounts of cpzA3H (%)”. The statistical analysis and bar graph preparation were performed using GraphPad Prism (version 10).

### In vitro reconstituted ubiquitination assay

An in vitro reconstituted ubiquitination assay was performed as previously reported, with slight modifications^35^. Briefly, Recombinant NEDD8, NEDD8 E1 NAE1-UBA3, and UBE2F proteins were purchased from R&D Systems. For in vitro neddylation of CUL5/RBX2, purified HIS6-tagged CUL5/RBX2 was neddylated in a buffer consisting of 25 mM Tris-HCl, pH 7.4, 100 mM NaCl, 10 mM MgCl2, and 1 mM ATP. The reaction was assembled by the sequential addition of 0.15 µM NEDD8 E1 NAE1-UBA3, 2 µM UBE2F, 24 µM NEDD8, and 12 µM HIS6-tagged CUL5/RBX2, incubated at 26 °C for 20 minutes, and then quenched with 10 mM DTT for 15 minutes on ice. The resulting products were purified using a HiLoad 26/600 Superdex 200 column and concentrated to 40 µM using an Amicon Ultra 30 K filter. Subsequently, in vitro reconstituted ubiquitination assays were conducted using a pulse-chase assay. Recombinant proteins Ubiquitin (Ub) WT, Ub mutant No Lysin (K0), and UBE1 were also obtained from R&D Systems. In the “pulse” reaction, Ub was thioester-linked to E2 (10 µM UbcH7) by incubating 15 µM Ub (WT or K0) and 300 nM UBE1 in 25 mM Tris-HCl, 100 mM NaCl, 2.5 mM MgCl2, 1 mM ATP, pH 7.4 for 10 min at 26 °C. The pulse reaction was quenched with 50 mM EDTA. For the “chase” step, the E2~Ub conjugate (2 µM) was added to a mixture containing 1 µM V2CBC WT, 1 µM NEDD8 ~ CUL5-RBX2, 1 µM cpzA3H, and 1 µM ARIH2. These reactions were performed at 26 °C for 30 min in 25 mM Tris-HCl, 100 mM NaCl, and 50 mM EDTA, pH 7.4. The reaction mixture was quenched with SDS sample buffer containing 15 µM DTT or flash-frozen on dry ice and stored at −80 °C until analysis. Ubiquitinated cpzA3H proteins were analyzed by SDS-PAGE and immunoblotting using anti-A3H rabbit antibodies (Novus Biologicals, cat # NBP1-91682).

### Identification of ubiquitinated lysine using mass spectrometry

Sample preparation: 600 µL of the in vitro ubiquitination mixture, containing cpzA3H protein at a concentration of 20 µg/mL, was divided into two samples. Both samples were digested with Trypsin using the standard in-solution digestion procedure. One sample was used for the enrichment of K-ɛ-GG peptides, following the protocol from the PTM-Scan Ubiquitin Remnant Motif (K-ɛ-GG) Kit (Cell Signaling Technology). The digested peptide mixtures, or the peptides after enrichment, were concentrated and desalted using C18 Ziptips (Millipore). The desalted enriched peptides were reconstituted in 16 µL of 0.1% formic acid, and 14 µL of peptide sample was analyzed by a 110-minute LC/MS/MS run. 2 μl of peptide sample was analyzed by 110-min LC/MS/MS run.

The LC/MS/MS analysis of tryptic peptides for each sample was performed sequentially using a Thermo Scientific Orbitrap Exploris 480 Mass Spectrometer and a Thermo Vanquish Neo RSLCnano System. Peptides from trypsin digestion were loaded onto a peptide trap cartridge at a flow rate of 5 μL/min. The trapped peptides were eluted onto a reversed-phase Easy-Spray Column PepMap RSLC, C18, 2 μM, 100 A, 75 μm × 250 mm (Thermo Fisher Scientific) using a linear gradient of acetonitrile (3-36%) in 0.1% formic acid. The elution duration was 60 min at a flow rate of 0.3 μl/min. Eluted peptides from the Easy-Spray column were ionized and sprayed into the mass spectrometer, using a Nano Easy-Spray Ion Source (Thermo Fisher Scientific) under the following settings: spray voltage, 1.6 kV, Capillary temperature, 275 °C. Other settings were empirically determined. Raw data files were searched against the HIV-1 or HIV2 protein sequence database and the target protein sequences database using the Proteome Discoverer 2.5 software (Thermo, San Jose, CA) based on the SEQUEST algorithm. Carbamidomethylation (+57.021Da) of cysteines was set as a fixed modification, and Oxidation/+15.995Da (M), Deamidation/+0.984Da (N, Q), and Ubiquitination/+114.043Da (K) were set as dynamic modifications. The minimum peptide length was specified as 5 amino acids. The precursor mass tolerance was set to 15 ppm, whereas fragment mass tolerance was set to 0.05 Da. The maximum false peptide discovery rate was specified as 0.05. The resulting Proteome Discoverer Report (Supplementary Table 2) contains all assembled proteins with peptide sequences and peptide spectrum match counts (PSM#) and MS1 peak area. Mass spectrometry raw data are provided as Supplementary Data 1.

### Wheat germ cell-free protein synthesis

cDNAs encoding HIV-2 Vif (NMC716, NMC842, ROD10, and UC1), SIVmac Vif, HIS-CBFß were amplified by PCR and subcloned into the pEU vector, specifically designed for the wheat germ cell-free system (CellFree Sciences). The cell-free translation reactions (220 μL) were conducted at 16 °C for 18 h in a 96-well plate using WEPRO7240 kit according to the manufacturer’s protocols (CellFree Sciences). The crude recombinant proteins were analyzed via SDS-PAGE, followed by Coomassie Brilliant Blue (CBB) staining.

### Reporting summary

Further information on research design is available in the Nature Portfolio Reporting Summary linked to this article.

## Supplementary information

Supplementary Information

Description of Additional Supplementary Files

Supplementary Data 1

Supplementary Data 2

Reporting Summary

Transparent Peer Review file

## Source data

Source Data

## Data Availability

Atomic coordinates for cpzA3H–V2CBC structure generated in this study was deposited in the Protein Data Bank under accession code 11ON. Unsharpened cryo-EM density map and half-maps for cpzA3H–V2CBC generated in this study was deposited in the Electron Microscopy Data Bank under accession code EMD-75888 [https://www.ebi.ac.uk/emdb/ EMD-75888]. Other structures used in this study include:9E93 https://doi.org/10.2210/pdb9E93/pdb 4n9f https://doi.org/10.2210/pdb4n9f/pdb, 5z98 https://doi.org/10.2210/pdb5Z98/pdb, 7b5m https://doi.org/10.2210/pdb7B5M/pdb,7b5l https://doi.org/10.2210/pdb7B5L/pdb,7oni https://doi.org/10.2210/pdb7ONI/pdb,8fvi https://doi.org/10.2210/pdb8FVI/pdb,Mass spectrometry raw data are provided as Supplementary Data 1. Sequences of DNA primers used in this study are provided as Supplementary Data 2. Source data are provided with this paper.
