# Supplementary material for: Divergent interfaces and convergent outcome in the structural basis of HIV-2 Vif antagonism of APOBEC3H
Source: Nat Commun. 2026 Oct 7;17:11437. doi: 10.1038/s41467-026-78197-2 (PMC13737106; doi:10.1038/s41467-026-78197-2)
Supplement: Supplementary file 1 [file 41467_2026_78197_MOESM1_ESM.pdf]

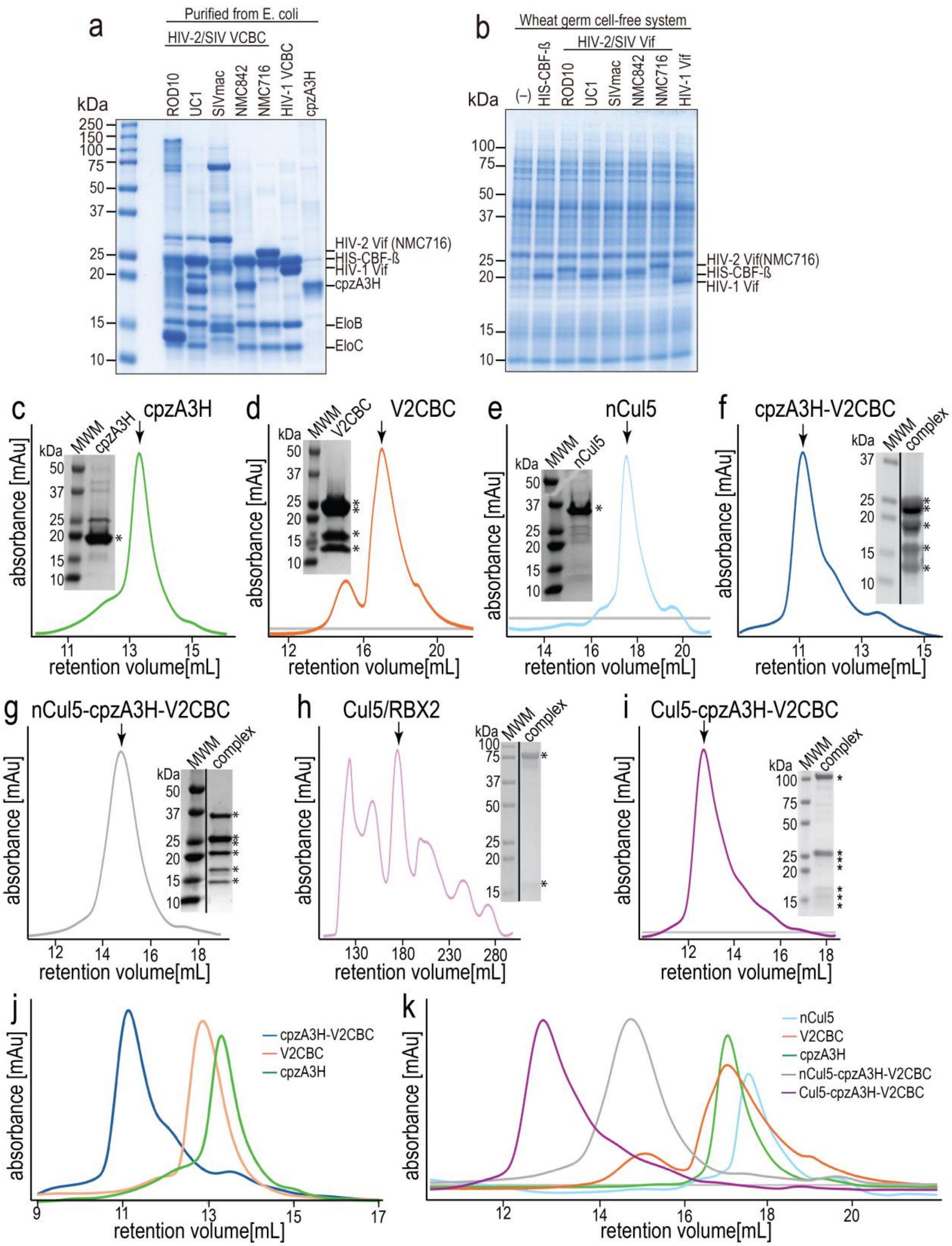

a
Purified from E. coli
HIV-2/SIV VCBC
kDa
ROD10
UC1
SIVmac
NMC842
NMC716
HIV-1 VCBC
cpzA3H
HIV-2 Vif (NMC716)
HIS-CBF-ß
HIV-1 Vif
cpzA3H
EloB
EloC
b
Wheat germ cell-free system
HIV-2/SIV Vif
(–)
HIS-CBF-ß
HIV-1 Vif
HIV-2 Vif(NMC716)
c
cpzA3H
d
V2CBC
e
nCul5
f
cpzA3H-V2CBC
g
nCul5-cpzA3H-V2CBC
h
Cul5/RBX2
i
Cul5-cpzA3H-V2CBC
MWM
complex
absorbance [mAu]
retention volume[mL]
j
k

Supplementary Figure 1. ***Purification of the HIV-2/SIVmac VCBC complex.***

(**a**) Coomassie-stained (CBB) SDS-PAGE analysis of purified HIV-2/SIV VCBC complexes. The tetrameric VCBC complexes containing each strain Vif (ROD10: HIV-2 group A strain, UC1: HIV-2 group B strain, SIVmac239: a rhesus macaque-adapted simian immunodeficiency viral isolate, NMC842: HIV-2 CRF01_AB strain, and NMC716: HIV-2 CRF01_AB strain) were co-expressed in *Escherichia coli* expression system. Each VCBC complex was purified via tandem affinity and cation-exchange chromatography. Purified proteins were separated using SDS–PAGE and stained with CBB. The HIV-1 VCBC complex (lane 6) and cpzA3H protein (lane 7) were used as control.

(b) Production of HIV-2/SIV Vif protein using a wheat germ cell-free system. Recombinant protein was synthesized using the bilayer method with the WEPRO7240 kit, and a 1.5-μl aliquot of the crude sample was separated by SDS-PAGE. Control reaction products with no mRNA added (–) (lane 1), HIS-CBFß (lane 2), and HIV-1 Vif (lane 7) were used as controls.

(c-k) Size-exclusion chromatograms and SDS-PAGE analyses of the complexes discussed in this study. (**c**) Chromatogram of purified cpzA3H, with an inset showing CBB SDS-PAGE of the peak fraction (indicated by arrow); column: Superdex 200 Increase 10/300 GL column. **(d)** Chromatogram of the purified HIV-2 NMC716 VCBC complex (V2CBC), with peak fraction (arrow) shown in the CBB SDS-PAGE inset; column: Superdex 200 Increase 10/300 GL column. **(e)** Chromatogram of purified N-terminal Cul5 (nCul5, 10-320 a.a.), with peak fraction (arrow) shown in the CBB SDS-PAGE inset); column: Superose™ 6 10/300 GL. **(f)** Chromatogram of a 1:1 molar mixture of cpzA3H and V2CBC, with peak fraction (arrow) shown in the CBB SDS-PAGE inset indicating all

five proteins corresponding to the cpzA3H-V2CBC complex. Peak fractions were used to set up cryo-EM grids. (**g**) Chromatogram of a 1:1:1 molar mixture of cpzA3H, nCul5 and V2CBC, with peak fraction (arrow) shown in the CBB SDS-PAGE inset indicating all six proteins corresponding to the nCul5-cpzA3H-V2CBC complex); column: Superose™ 6 10/300 GL. Peak fractions were used to set up cryo-EM grids. (**h**) Chromatogram of purified Cul5/RBX2, with peak fraction (arrow) shown in the CBB SDS-PAGE inset; column: Hiload 26/600 Superdex 200. **(i)** Chromatogram of a 1:1:1 molar mixture of cpzA3H, Cul5/RBX2 and V2CBC, with peak fraction (arrow) shown in the CBB SDS-PAGE inset indicating all seven proteins corresponding to the Cul5/RBX2-cpzA3H-VCBC complex); column: Superose™ 6 10/300 GL. Peak fractions were used to set up cryo-EM grids. (**j**) Superimposed chromatograms of cpzA3H alone (green), V2CBC alone (orange), and the cpzA3H-V2CBC complex (f, navy); column: Superdex 200 Increase 10/300 GL column. (**k**) Superimposed chromatograms from (**c**), (**d**), **(e)**, **(g)**, and (**i**), indicating cpzA3H alone (green), V2CBC alone (orange), nCul5 (sky blue), the nCul5-cpzA3H-V2CBC complex (grey), and the Cul5/RBX2-cpzA3H-V2CBC complex (violet); column: Superose™ 6 10/300 GL.

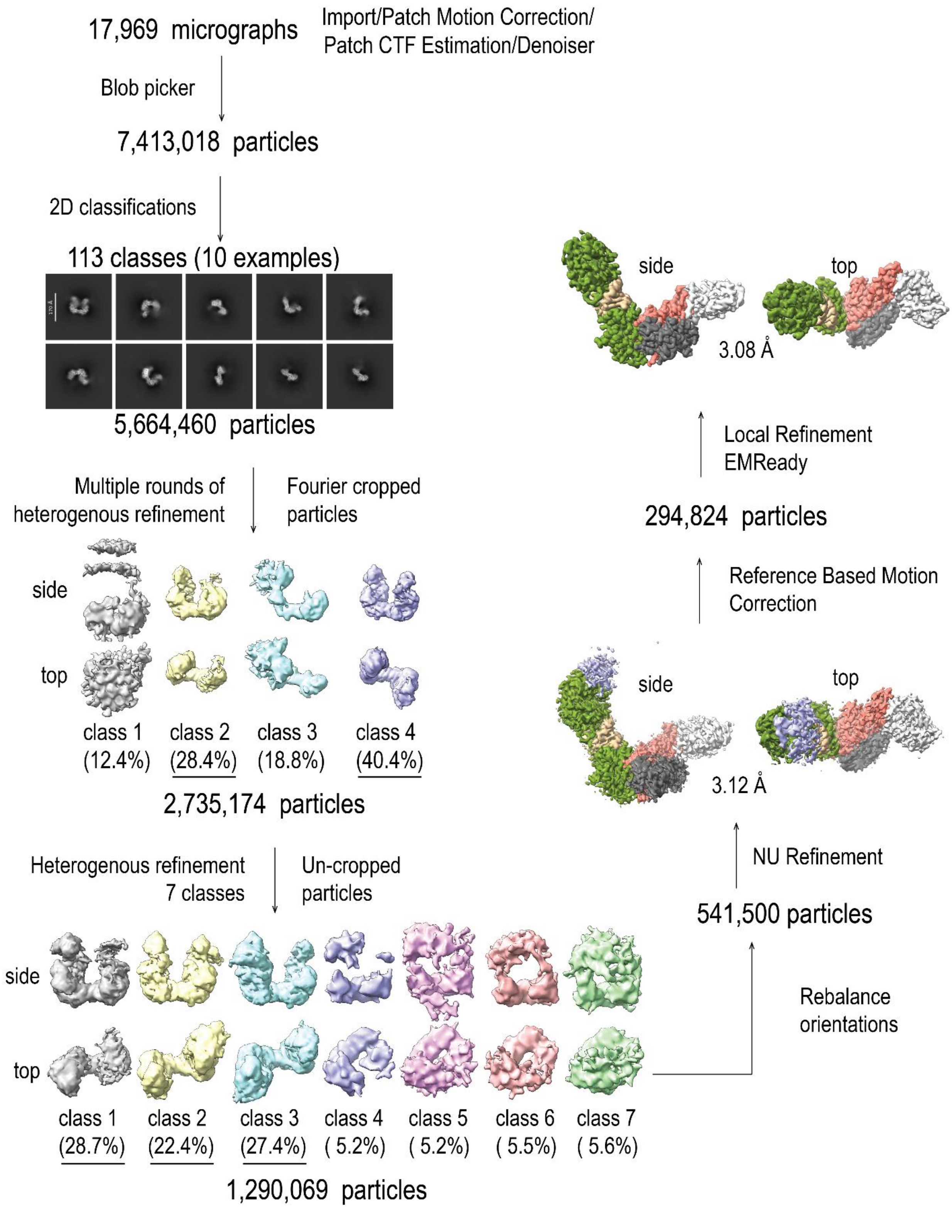

Supplementary Figure 2. ***Cryo-EM image processing workflow.***

Movie data were collected using a K3 direct electron detector in counting mode on a Talos Arctica microscope operating at 200 kV. A total of 7,413,018 Fourier-cropped particles were initially extracted from 17,969 motion-corrected, dose-weighted micrographs. Following 2D classification, classes lacking defined structural features (e.g., ice contamination or carbon edge artifacts) were discarded, resulting in 5,664,460 particles retained across 113 classes. The relatively high number of accepted particles reflects strong preferred orientation in the dataset, and less populated views were intentionally retained.

After heterogeneous refinement, classes corresponding to complexes of the expected size were selected, and 2,735,174 particles were re-extracted from the original micrographs without Fourier cropping. A subsequent round of heterogeneous refinement yielded 1,290,069 particles, which were subjected to orientation rebalancing, resulting in 541,500 particles. These particles were used for reference-based motion correction, followed by local refinement of 294,824 particles, yielding a reconstruction at 3.08 Å resolution, as determined by the gold-standard Fourier shell correlation (FSC = 0.143). The final map was subjected to post-processing using EMready[1] to improve interpretability.

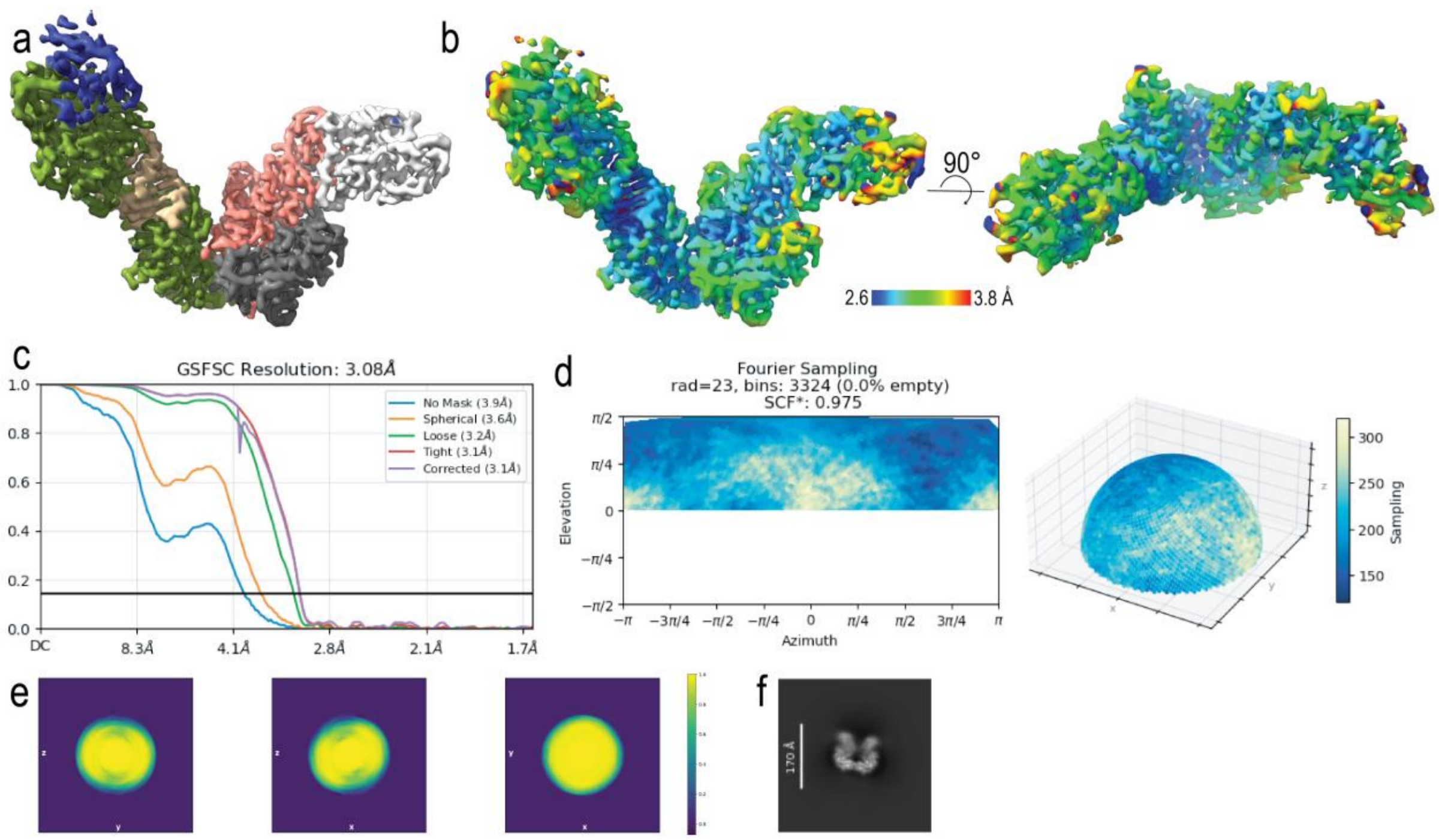

Supplementary Figure 3. ***Resolution analysis of the cpzA3H-V2CBC complex*****.**

(a) Cryo-EM density map of the cpzA3H–V2CBC complex colored by component: cpzA3H (green), dsRNA (pale yellow), CBFβ (dark grey), Vif (salmon), and Elongin B/C (light grey). Additional density (purple) may correspond to a second copy of the V2CBC complex bound to cpzA3HB and was masked out in the main figures. **(b)** Local resolution mapped onto the cryo-EM density, highlighting higher resolution at the cpzA3H–Vif interface. **(c)** Global resolution estimation based on the gold-standard Fourier shell correlation (FSC) using the 0.143 criterion. **(d)** Angular distribution of particle orientations. **(e)** 3DFSC volume central slice clipped at 2.992 Å shows residual anisotropy in one direction[2]. **(f)** 2D class shows two copies of V2CBC bound to a A3H dimer; despite being removed from the final reconstruction, the residual signal shows up as extra density (blue; panel (a)).

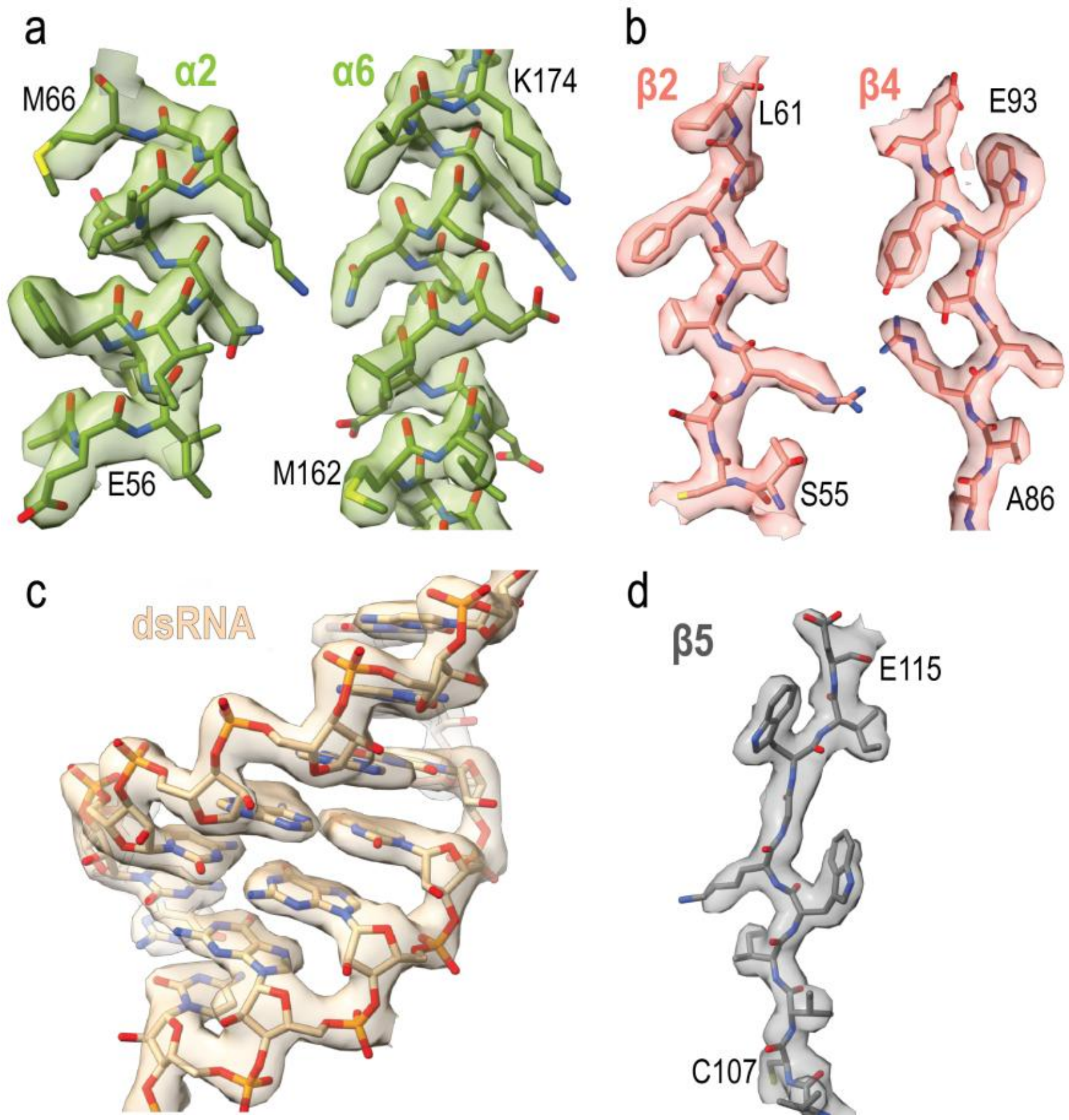

Supplementary Figure 4. ***Model to map fit.***

Representative secondary structures of (a) cpzA3H$_A$, (b)Vif, (c) dsRNA, and (d) CBFβ are shown with the corresponding cryo-EM densities.

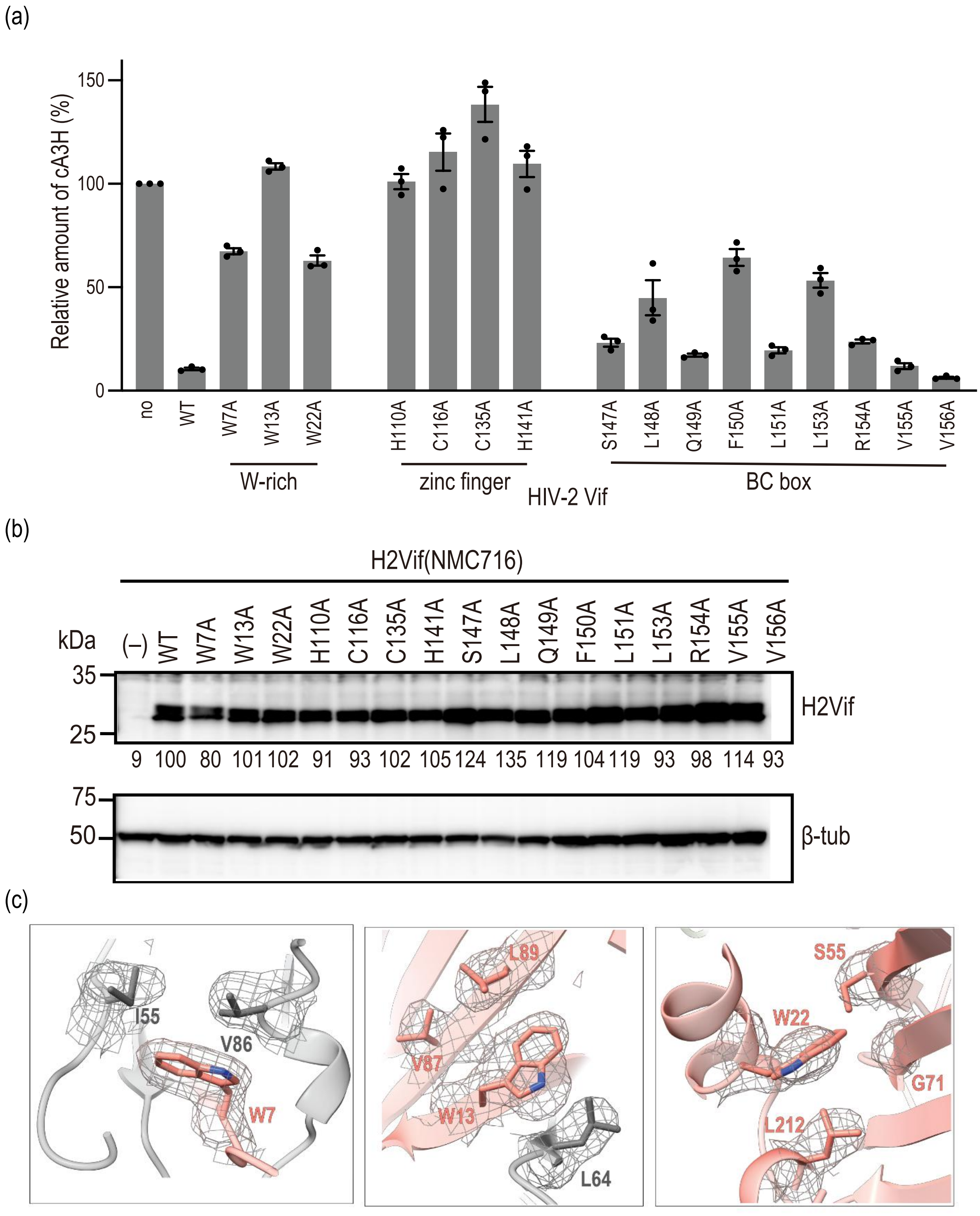

Supplementary Figure 5. ***HiBiT alanine substitution of HIV-2 Vif residues required for CUL5 E3 ligase assembly.***

(a) Alanine substitutions were introduced individually into HIV-2 Vif residues. Each mutant was co-expressed with cpzA3H in HEK293T cells, and steady-state cpzA3H levels were quantified by HiBiT assay. Degradation activity is presented as the percentage reduction in cpzA3H levels relative to the no-Vif control (no), normalized to wild-type HIV-2 Vif (WT). Data represent mean ± SEM from three independent experiments, with individual data points are overlayed on the corresponding bars. Vif residues structurally important for CUL5 E3 ligase assembly, including the N-terminal tryptophan-rich region, the zinc finger, and the BC-box, exhibit markedly reduced cpzA3H degradation. (b) HIV-2 Vif expression levels for wild-type and mutant variants. HEK293T cells were transfected with expression constructs for HIV-2 Vif wild-type (WT) and mutants, and intracellular protein levels were analyzed 40 hours post-transfection by Western blot using anti-HIS antibody. β-tubulin served as a loading control. Band intensities were quantified by densitometry and normalized to β-tubulin; expression levels are displayed as a percentage of wild-type Vif set at 100%. Most mutants maintained >90% of wild-type Vif expression levels; only the W74A mutant showed reduced expression at approximately 80%. These results demonstrate that observed impairment of A3H degradation by Vif mutants reflects direct functional defects in Vif rather than secondary effects on protein expression or stability. (c) Close-up view of the HIV-2 Vif N-terminal tryptophan cluster and its role in Vif–CBFβ interface stabilization. W7 engages CBFβ I55 and V86, while W13 contacts CBFβ L64 and Vif V87/L89, together forming an intermolecular β-sheet. W22 stabilizes the hydrophobic core between α-helices α1 and α6 of the Vif scaffold. Cryo-EM density is displayed as a transparent mesh. These interactions collectively stabilize the structural integrity of the N-terminal Vif–CBFβ interface.

(a)

sequence alignment between cpzA3H and hA3H hapII, with focus on amino acid differences

```
hA3H      1   MALLTAETFR LQFNNKRRLR RPYYPRKALL CYQLTPQNGS TPTRGYFENK KKCHAEICFI   60
cpzA3H    1   MALLTAETFR LQFNNRRRLR RPYYPRKALL CYQLTPQNGS TPTRGYFENK KKCHAEICFI   60

hA3H     61   NEIKSMGLDE TQCYQVTCYL TWSPCSSCAW ELVDFIKAHD HLNLRIFASR LYYHWCKPQQ  120
cpzA3H   61   NEIKSMGLDE TQCYQVTCYL TWSPCSSCAW KLVDFIQAHD HLNLRIFASR LYYHWCKPQQ  120

hA3H    121   DGLRLLCGSQ VPVEVMGFPE FADCWENFVD HEKPLSFNPY KMLEELDKNS RAIKRRLDRI  180
cpzA3H  121   EGLRLLCGSQ VPVEVMGLPE FNDCWENFVD HEKPLSFDPC KMLEELDKNS RAIKRRLERI  180

hA3H    181   KQS 183
cpzA3H  181   KQS 183
```

(b)

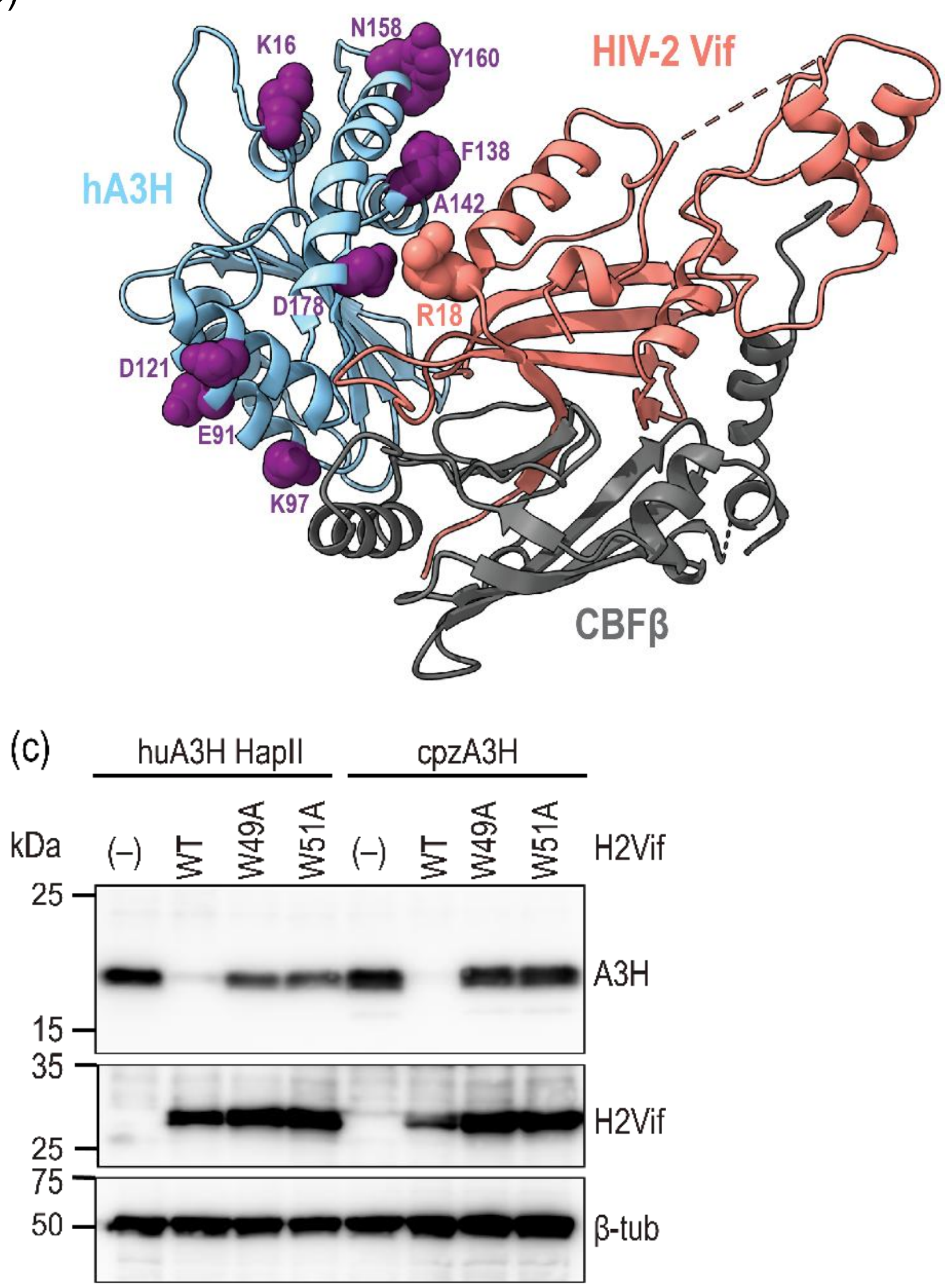

Supplementary Figure 6 - Functional conservation of the HIV-2 Vif–A3H interaction between chimpanzee and human A3H haplotype II

(a) Sequence alignment of cpzA3H and human A3H haplotype II (hapII). Residues in human A3H hapII that differ from cpzA3H are highlighted in blue. Residues in cpzA3H that interact with HIV-2 Vif are highlighted in salmon. (b) Ribbon diagram of human A3H haplotype II (blue) superimposed on cpzA3H. Amino acid differences are shown as purple spheres; the only interfacial difference is at position 178 (cpzA3H E178 vs. human A3H haplotype II D178), a conservative substitution. Vif R18, which contacts this position, is shown as salmon spheres. (c) HIV-2 Vif-mediated degradation of human A3H haplotype II. Consistent with the high sequence conservation at the Vif–A3H interface, HIV-2 Vif efficiently degrades human A3H haplotype II, while W49A/W51A substitutions markedly attenuate degradation. HEK293T cells were transfected with the indicated constructs, and lysates were analyzed by Western blot with antibodies against A3H, HIV-2 Vif, and β-tubulin (loading control). WT, wild-type Vif; (−), empty vector control.

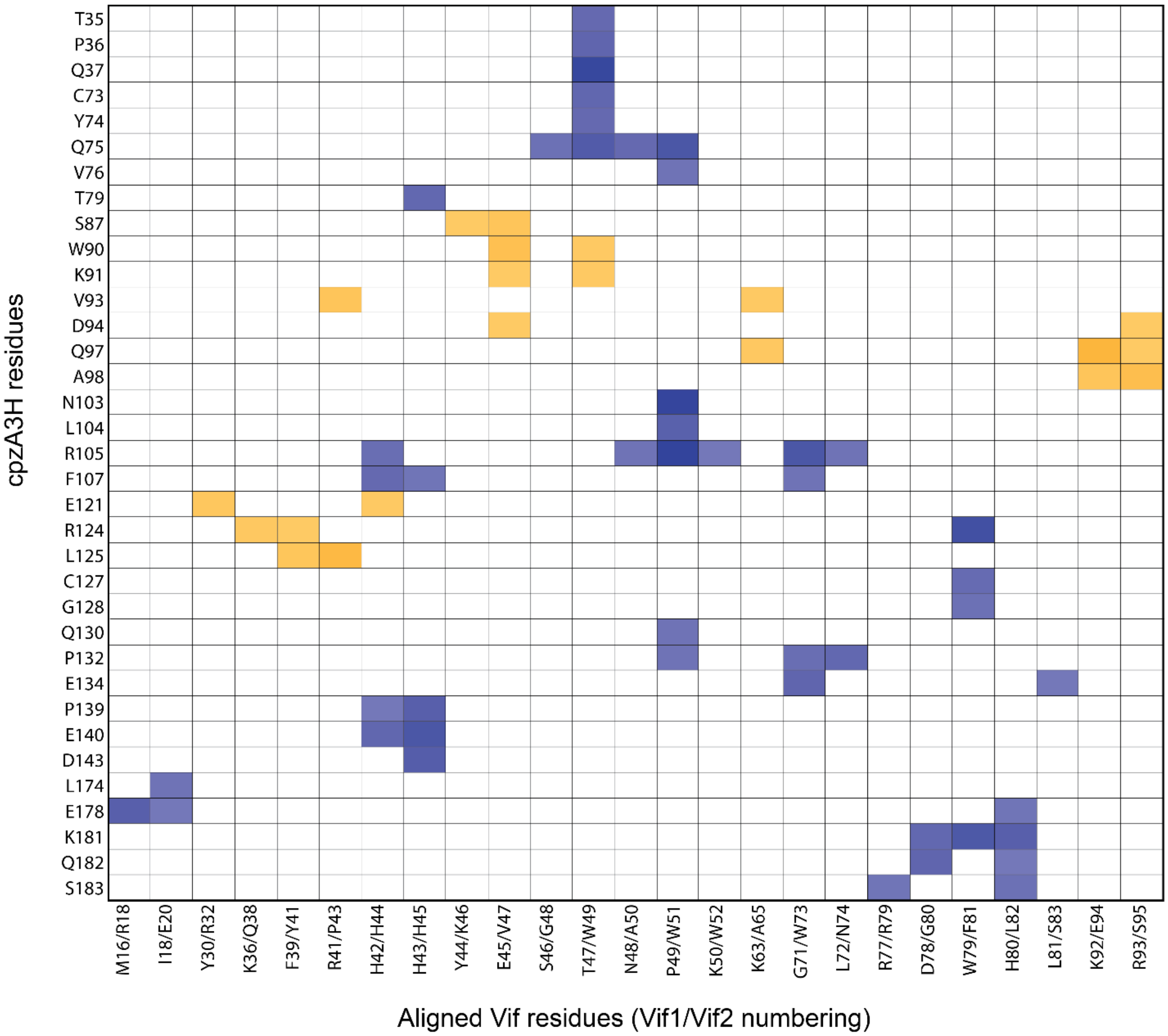

Supplementary Figure 7. ***Contact heatmap of Vif–cpzA3H interactions.***

A contact heatmap summarizing interactions between Vif molecules and cpzA3H residues, using a distance cutoff of 5 Å. cpzA3H residues (y-axis) are plotted against aligned HIV-1 and HIV-2 Vif residues (x-axis). HIV-2-specific contacts are shown in blue and HIV-1-specific contacts in orange. The largely non-overlapping interaction patterns highlight distinct binding interfaces used by HIV-1 and HIV-2 Vif.

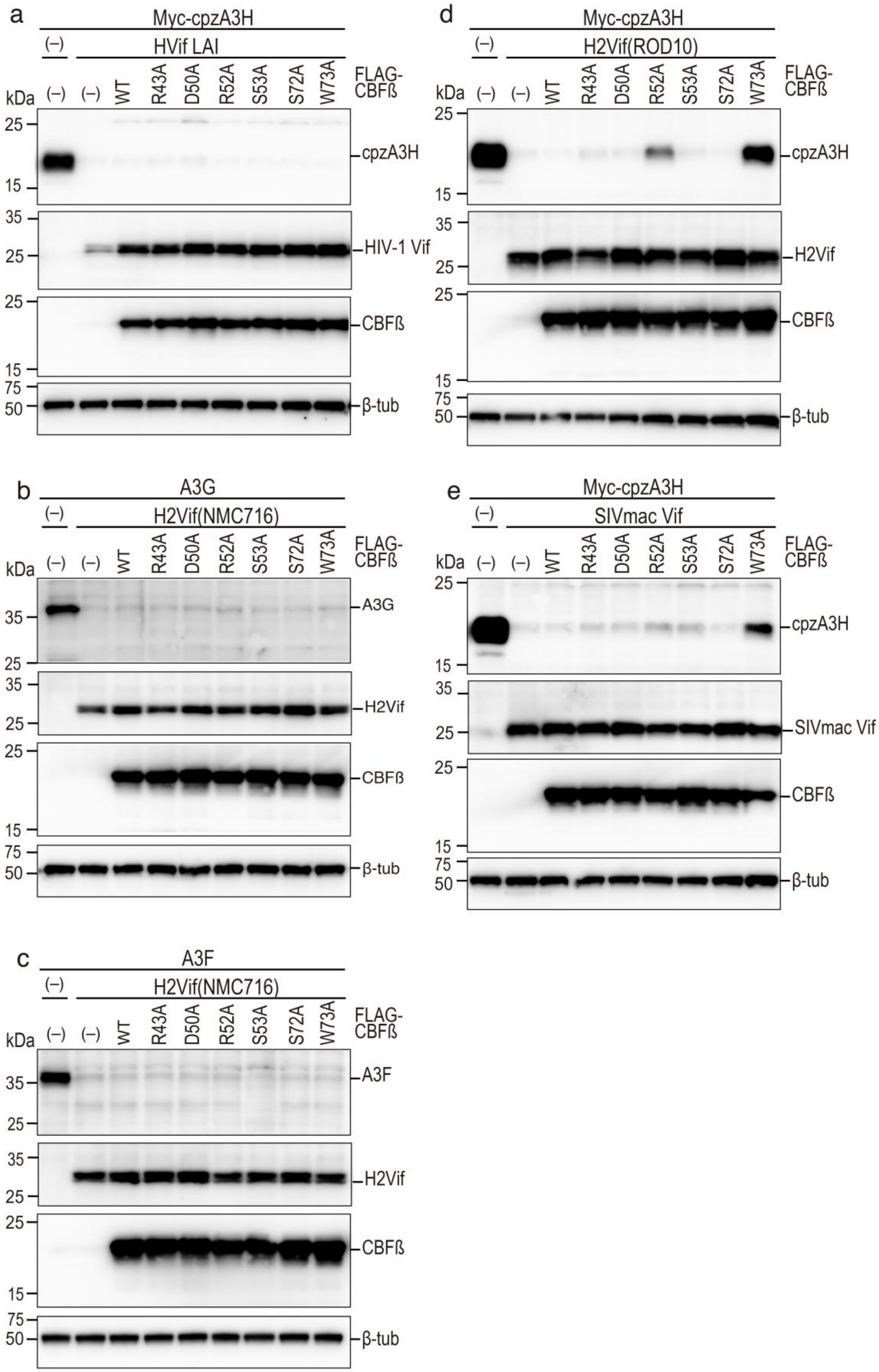
a
Myc-cpzA3H
(–)
HVif LAI
kDa
(–)
(–)
WT
R43A
D50A
R52A
S53A
S72A
W73A
FLAG-CBFß
25
15
cpzA3H
35
25
HIV-1 Vif
25
15
CBFß
75
50
β-tub
b
A3G
(–)
H2Vif(NMC716)
kDa
(–)
(–)
WT
R43A
D50A
R52A
S53A
S72A
W73A
FLAG-CBFß
35
25
A3G
35
25
H2Vif
25
15
CBFß
75
50
β-tub
c
A3F
(–)
H2Vif(NMC716)
kDa
(–)
(–)
WT
R43A
D50A
R52A
S53A
S72A
W73A
FLAG-CBFß
35
25
A3F
35
25
H2Vif
25
15
CBFß
75
50
β-tub
d
Myc-cpzA3H
(–)
H2Vif(ROD10)
kDa
(–)
(–)
WT
R43A
D50A
R52A
S53A
S72A
W73A
FLAG-CBFß
25
15
cpzA3H
35
25
H2Vif
25
15
CBFß
75
50
β-tub
e
Myc-cpzA3H
(–)
SIVmac Vif
kDa
(–)
(–)
WT
R43A
D50A
R52A
S53A
S72A
W73A
FLAG-CBFß
25
15
cpzA3H
35
25
SIVmac Vif
25
15
CBFß
75
50
β-tub

Supplementary Figure 8. ***Effect of CBFβ amino acid substitution at the CBFβ-cpzA3H interface on HIV-1, HIV-2, SIVmac Vif-mediated A3F, A3G, and A3H degradation.***

Individual FLAG–CBFβ residues buried at the CBFβ–HIV-2 Vif interface (**Fig. 5**) were substituted with alanine. Degradation of Myc-tagged cpzA3H was assessed in the presence of HIV-1 LAI Vif–MycHIS (a), HIV-2 Vif (ROD10)–MycHIS (d), or SIVmac Vif–MycHIS (e). Degradation of A3G (b) or A3F (c) was assessed in the presence of HIV-2 Vif (NMC716)–MycHIS. Intracellular levels of cpzA3H, A3F, A3G, Vif, and CBFβ were analyzed 40 h post-transfection by western blotting using anti-Myc, anti-HIS, and anti-FLAG monoclonal antibodies, and anti-A3G and anti-A3F polyclonal antibodies. β-tubulin (β-Tub) was used as a loading control. Stability of A3H (a, d, e), A3G (b), and A3F (c) in the absence of both Vif and exogenous FLAG–CBFβ (lane 1), in the presence of Vif alone (lane 2), and in the presence of both Vif and wild-type FLAG–CBFβ (lane 3) served as controls. Data are representative of three independent experiments.

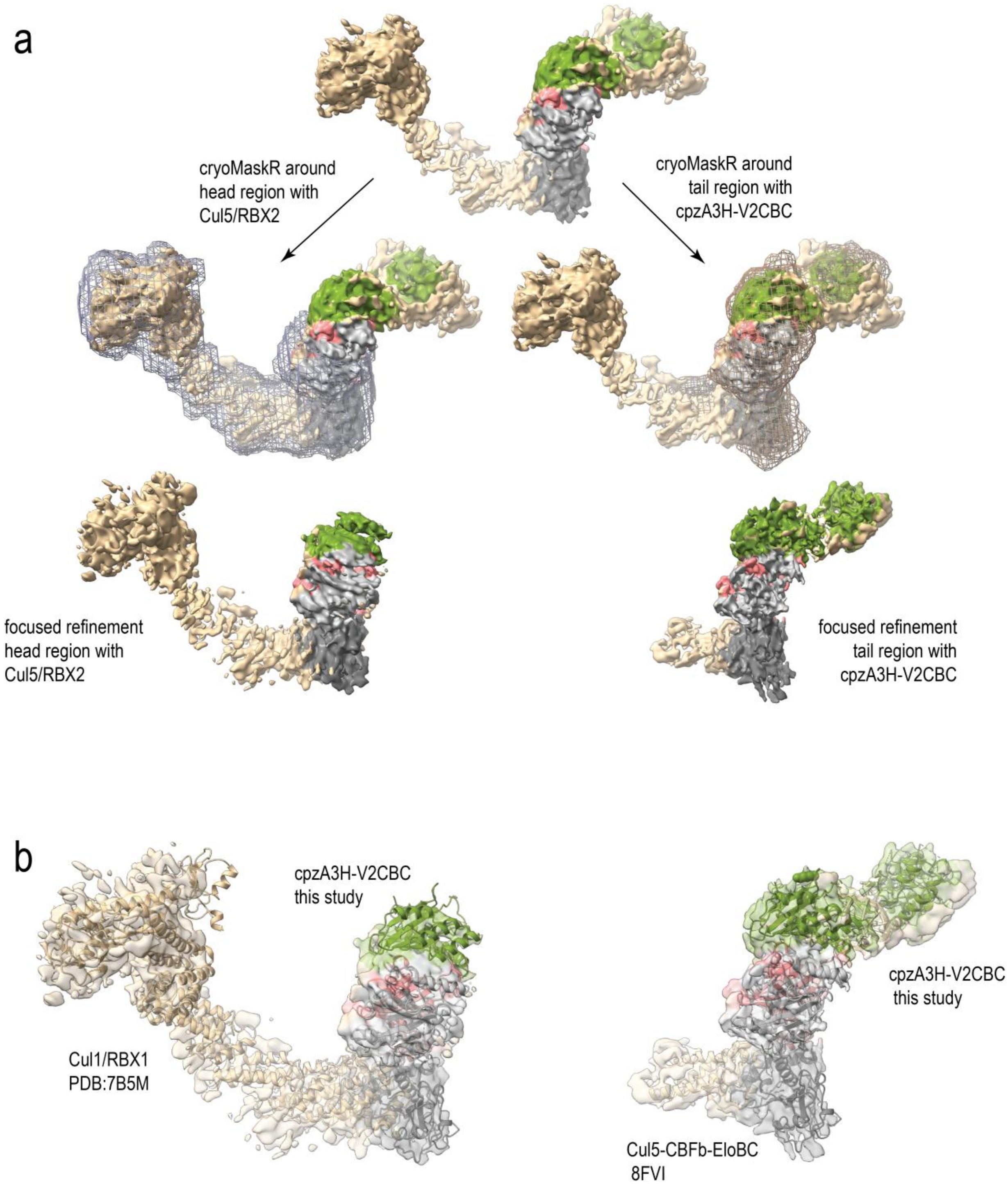
a
cryoMaskR around
head region with
Cul5/RBX2
cryoMaskR around
tail region with
cpzA3H-V2CBC
focused refinement
head region with
Cul5/RBX2
focused refinement
tail region with
cpzA3H-V2CBC
b
cpzA3H-V2CBC
this study
Cul1/RBX1
PDB:7B5M
cpzA3H-V2CBC
this study
Cul5-CBFb-EloBC
8FVI

Supplementary Figure 9. ***Cryo-EM analysis of Cul5/RBX2-cpzA3H-V2CBC complex.***

(a) Cryo-EM density map of the Cul5–RBX2–cpzA3H–V2CBC complex colored by component: Cul5–RBX2 (tan), cpzA3H (green), dsRNA (pale yellow), CBFβ (dark grey), Vif (salmon), and Elongin B/C (light grey). The overall map quality is limited due to preferred particle orientations, prompting focused refinement of masked regions. Masked (lower panel) were generated using cryoMaskR[3] and are shown is colored mesh.

(b) Focused refinement of the head (Cul5–RBX2) and tail (cpzA3H–V2CBC) regions yielded maps of improved quality, facilitating interpretation and fitting of available structural models (as shown).

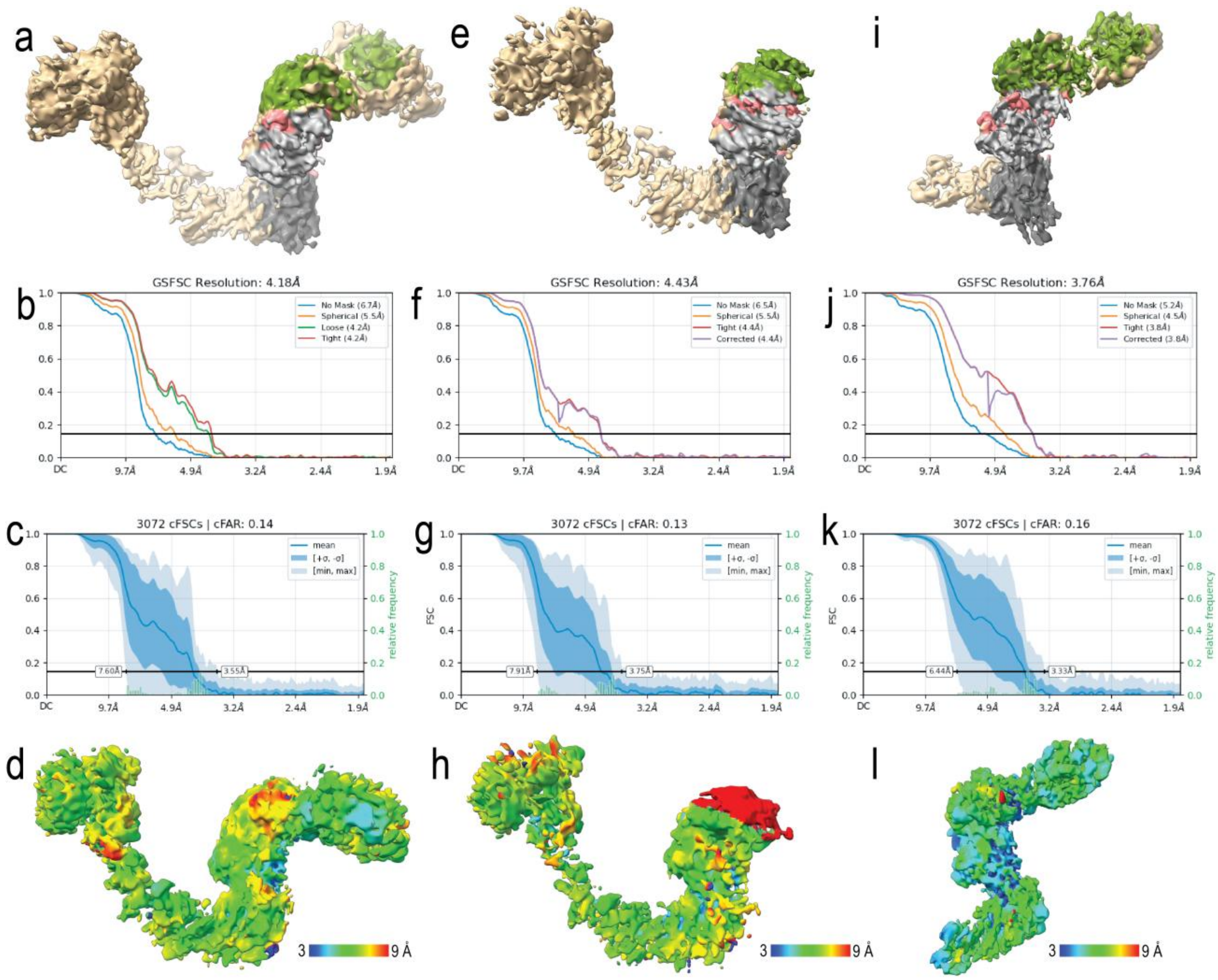

Supplementary Figure 10. ***Resolution analysis of the cpzA3H-V2CBC-Cul5/RBX2 complex*.**

(a–d) Full-length cpzA3H–V2CBC–Cul5/RBX2 complex. **(e–h)** Focused reconstruction on the Cul5/RBX2 subcomplex. **(i–l)** Focused reconstruction on the cpzA3H–V2CBC subcomplex.**(a, e, i)** Cryo-EM density maps colored by component: cpzA3H (green), dsRNA (pale yellow), CBFβ (dark grey), Vif (salmon), Elongin B/C (light grey) and Cul5/RBX2 (tan). **(b, f, j)** Gold-standard Fourier shell correlation (FSC) curves used for global resolution estimation at the 0.143 criterion. **(c, g, k)** Orientation distribution analysis

using the canonical FSC area ratio (cFAR), indicating preferred particle orientations in all reconstructions. **(d, h, l)** Local resolution mapped onto the cryo-EM density.

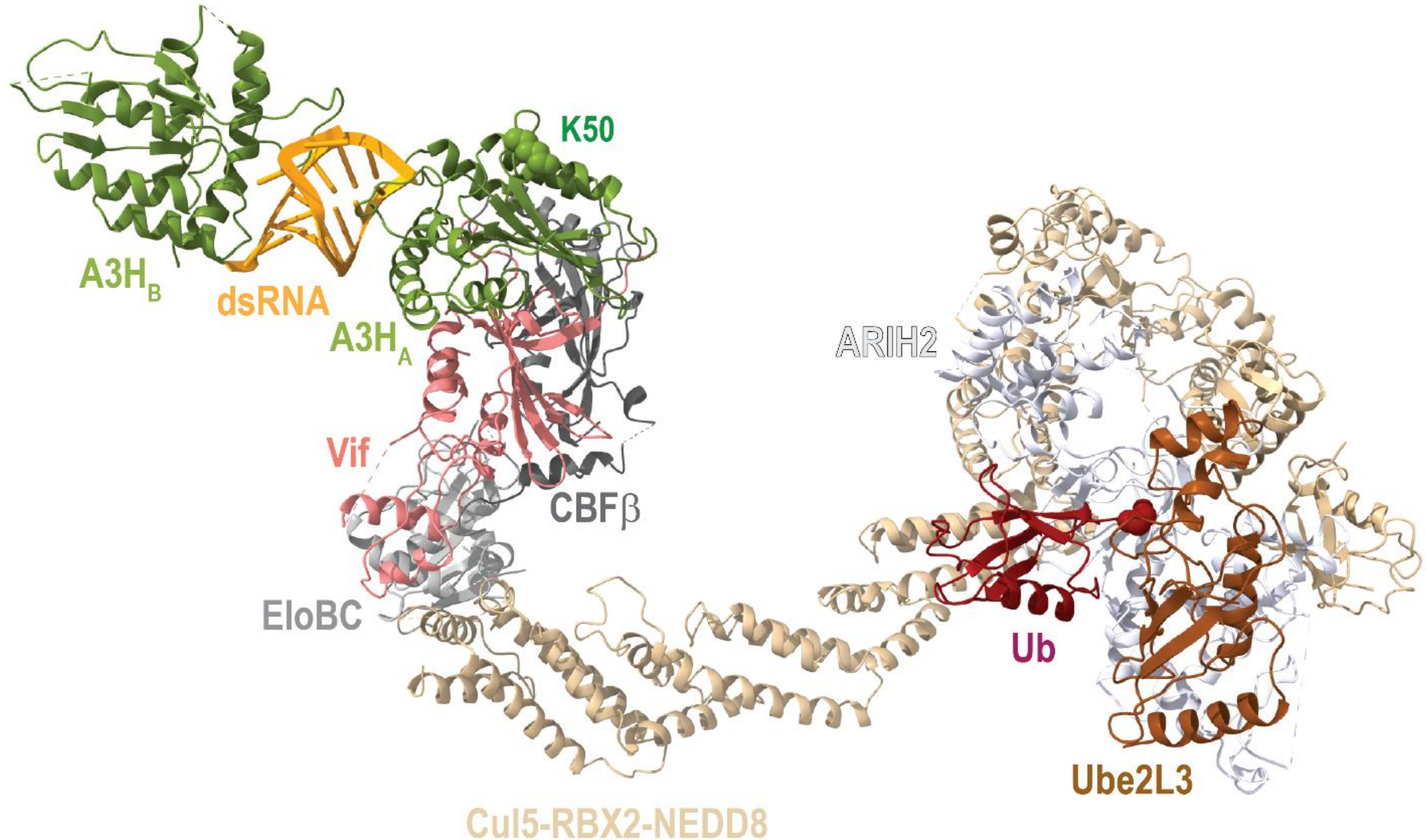

Supplementary Figure 11. Mechanistic model of cpzA3H ubiquitination by the HIV-2 Vif–CUL5 E3 ligase complex, with ARIH2 in Transition State 1.

Composite model of the complete ubiquitination machinery assembled on cpzA3H, built by integrating cryo-EM structures determined in this study with components from PDB entries 7B5L, 7ONI, and 8FVI. The model is shown in cartoon representation with components colored as follows: A3H subunit A ($A3H_A$) and A3H subunit B ($A3H_B$), green; dsRNA, orange; HIV-2 Vif, salmon; CBFβ, dark grey; ElonginB/C (EloBC), light grey; Cul5–RBX2–NEDD8, beige; ARIH2, lavender; Ube2L3, brown; and ubiquitin (Ub), maroon. The C-terminal glycine of ubiquitin (Gly76) and the target lysine of cpzA3H (K50) are shown as spheres. This model depicts the ARIH2 primed with UBE2L3 in Transition State 1[4], representing the initiation of ubiquitin transfer prior to mono-ubiquitination of $A3H_A$.

1. He, J., Li, T. & Huang, S.Y. Improvement of cryo-EM maps by simultaneous local and non-local deep learning. *Nat Commun* **14**, 3217 (2023).
2. Tan, Y.Z. et al. Addressing preferred specimen orientation in single-particle cryo-EM through tilting. *Nat Methods* **14**, 793–796 (2017).
3. Calthorpe-Byrne, M.J. cryomaskR: v0.1. Available at https://github.com/mcalbyrne/cryomaskr/releases/tag/v1.0.1.
4. Horn-Ghetko, D. et al. Ubiquitin ligation to F-box protein targets by SCF-RBR E3-E3 super-assembly. *Nature* **590**, 671–676 (2021).
