# Supplementary material for: Divergent interfaces and convergent outcome in the structural basis of HIV-2 Vif antagonism of APOBEC3H
Source: Nat Commun. 2026 Oct 7;17:11437. doi: 10.1038/s41467-026-78197-2 (PMC13737106; doi:10.1038/s41467-026-78197-2)
Supplement: Supplementary file 2 [file 41467_2026_78197_MOESM2_ESM.pdf]

Description of Additional Supplementary Files

File name: Supplementary Data 1
Description: Mass spectrometry raw data identifying ubiquitinated lysine residues in chimpanzee APOBEC3H induced by HIV-2 Vif.

File name: Supplementary Data 2
Description: Sequences of DNA primers used in this study.
