# Supplementary material for: Divergent interfaces and convergent outcome in the structural basis of HIV-2 Vif antagonism of APOBEC3H
Source: Nat Commun. 2026 Oct 7;17:11437. doi: 10.1038/s41467-026-78197-2 (PMC13737106; doi:10.1038/s41467-026-78197-2)
Supplement: Supplementary file 4 [file 41467_2026_78197_MOESM6_ESM.pdf]

nature portfolio

# Peer Review File

# Divergent interfaces and convergent outcome in the structural basis of HIV-2 Vif antagonism of APOBEC3H

Corresponding Author: Dr Hiroshi Matsuo

**This file contains all reviewer reports in order by version, followed by all author rebuttals in order by version.**

Version 1:

Reviewer comments:

Reviewer #1

(Remarks to the Author)
The manuscript presented a quality and potentially impactful structural study that defines the structural molecular basis by which HIV-2 Vif antagonizes A3H. The 3.08 Å cryo-EM structure of the HIV-2 Vif–cpzA3H–CBFβ–EloBC complex reveals that HIV-2 Vif adopts a globally conserved Vif fold while engaging A3H through a binding surface that is strikingly distinct from HIV-1 Vif, despite converging on the same functional outcome of targeting A3H K50 for ubiquitination and degradation. The major strengths include: 1). high-quality cryo-EM structural analysis. The map quality appears sufficient for reliable side-chain assignment at the Vif/A3H interface, and the manuscript provides detailed structural interpretation of the intermolecular contacts. 2). Clear mechanistic distinction between HIV-1 and HIV-2 Vif recognition of A3H. The comparative analysis convincingly demonstrates that HIV-1 and HIV-2 Vif proteins engage largely non-overlapping surfaces on cpzA3H despite maintaining a conserved overall Vif fold. This is conceptually important and represents a compelling example of convergent evolution at the structural level. 3). Integration of structure with biochemical validation. The mutational analyses of both Vif and A3H support the structural model and identify key determinants of A3H degradation, particularly the critical contribution of the aromatic W49/W51 cluster in HIV-2 Vif. 4). Identification of a novel CBFβ/A3H interface. The discovery of a direct CBFβ/A3H interaction unique to the HIV-2 complex is particularly interesting. 5). Broad biological relevance: The manuscript addresses an important gap in understanding HIV-2 biology and provides a framework for studying lineage-specific Vif adaptation across primate lentiviruses.

Overall, this is a strong study that combines several approaches, including structural biology, mutagenesis, evolutionary analysis, and biochemical validation to address an important question in lentiviral host adaptation. The work significantly advances understanding of HIV-2 Vif, which remains much less characterized than HIV-1 Vif, and provides valuable mechanistic insight into convergent evolution of A3-mediated antagonism of HIV strains. However, several mechanistic conclusions would benefit from additional experimental support and clearer discussion of the limitations of the structural modeling. Addressing the points above would significantly strengthen the manuscript.

Major concerns
1. Physiological relevance to human A3H variants requires clearer treatment
The structural work relies primarily on chimpanzee A3H (cpzA3H), while the manuscript extrapolates conclusions to human A3H haplotypes. Because human A3H is highly polymorphic and exhibits major haplotype-dependent differences in stability and Vif susceptibility, the manuscript should more clearly define the limitations of extrapolating from cpzA3H to human A3H. Additional functional testing using at least one or two human A3H haplotypes, particularly haplotype II, would substantially strengthen the translational relevance of the study. If such experiments are not feasible, the Discussion should more explicitly acknowledge this limitation.
2. The proposed ubiquitination geometry appears largely model-driven
The manuscript proposes a structural model explaining how A3H K50 is positioned relative to the ubiquitination machinery. However, the full CUL5–RBX2-containing assembly appears to be substantially lower resolution and partially dependent on rigid-body fitting and homology modeling. The manuscript should more explicitly distinguish experimentally resolved structural features from inferred modeling. The Discussion and figure legends should clarify the uncertainty associated with the proposed ubiquitination geometry and avoid overstating mechanistic precision that is not directly supported by the cryo-EM density.

Additional technical and presentation issues

1. Even though the structure reveals a unique CBFβ/A3H interface in the HIV-2 complex, the HIV-1 A3F complexes also show CBFβ contacts (as discussed by the authors). Maybe tone down like: previously unobserved for A3H, or structurally distinct from HIV-1 A3F interactions, etc, to be on the safe side.
2. I cannot quantitatively assess complex formation shifts in Supplementary Figure 1.
3. The manuscript occasionally blurs HIV-2 vs SIVmac conclusions and discussion. I feel it would be easier for me to read it if there is a clearer distinction between directly tested systems (HIV-2 NMC716 with cpzA3H) and the inferred evolutionary generalizations.
4. The manuscript discusses severe preferred orientation that was partially alleviated by addition of nCUL5, although nCUL5 density is absent from the final reconstruction. Additional directional FSC or 3D FSC analysis would help readers evaluate any residual anisotropy in the final map.
5. Supplementary Figure 3 includes unexplained additional density proposed to correspond to a second V2CBC complex. The authors should clarify the basis for this interpretation and explain why the density was excluded from the final reconstruction.
6. The representative 2D class averages in Supplementary Figure 2 are difficult to evaluate because of their small size and low contrast. Enlarged representative class averages would improve presentation quality.
7. Some biochemical controls appear incomplete. For example, several Vif mutants in Supplementary Figure 5 appear to produce A3H levels above the no-Vif control. It would be important to show Vif expression levels for all mutants to distinguish direct functional effects from altered protein stability or expression.
8. The statement that W7 forms an intermolecular β-sheet with CBFβ and that W13/W22 stabilize a hydrophobic core is plausible but not sufficiently illustrated in the current figures. A close-up structural panel with corresponding density would strengthen this point.

Reviewer #2

(Remarks to the Author)
The manuscript, Divergent interfaces, convergent outcome: structural basis of HIV-2 Vif antagonism of APOBEC3H, reveals, through a variety of experiments, the critical structural interface and essential molecular interactions between the host restriction factor (A3H) and its viral antagonist (HIV-2 Vif). Unexpectedly, the analysis also uncovers a novel important interface between CBFbeta and A3H that has not been previously described. The biological significance of the findings is highlighted and verified in functional assays performed in tissue culture.

My comments for improvement of the manuscript focus exclusively on the functional assays and the corresponding author analysis and interpretation.

Primary suggestions:

1. The highlighting of the important interface between CBFbeta and A3H is a unique part of this submission (as noted by the authors in line 430) and, as such, I would recommend mentioning that finding in the abstract. The important binding events extend beyond Vif and A3 and this is an interesting result that ought to be clear to the reader from the outset!

2. In line 133, the phrase "…residues within the BC-box motif…" should be modified slightly because not all residues within this BC box contribute to functional Vif activity.

3. The importance of W49 and W51 in Vif antagonism of A3 family members has been previously described in different experimental settings. In particular, I think the 2014 publication from the Pathak lab ought to be cited somewhere in the manuscript:

Smith JL, Izumi T, Borbet TC, Hagedorn AN, Pathak VK. HIV-1 and HIV-2 Vif interact with human APOBEC3 proteins using completely different determinants. J Virol. 2014 Sep 1;88(17):9893-908. doi: 10.1128/JVI.01318-14. Epub 2014 Jun 18. PMID: 24942576; PMCID: PMC4136346.

4. Figure 5f results from a meticulous and well-planned experiment. However, the degradation of cpzA3H in the absence of transfected CBFbeta is glaring. While the authors do offer a reasonable explanation for the observation in the figure legend, I would recommend moving the explanation to the main body of the narrative to ensure clarity for the reader.

5. In Figure 5g, it is somewhat debatable whether the C73A/D100A substitution confers significant protection from degradation. These bands could be quantitated with ImageQuant if the argument is important enough to the conclusions.

Minor suggestions:

1. In the abstract, lines 31-32, "…HIV-2 and HIV-1 Vif both target A3H lysine 50…", as well as line 367, "…HIV-2 Vif primarily targets K50…" could be worded better for clarity. The Vif protein targets A3H for degradation by establishing a direct interaction that results in the accessibility of lysine 50. Vif does not really target an individual amino acid as the authors elegantly show with their extensive cryo-EM structure work.

2. What is the (functional/structural) relevance of the hydrophobic core described in line 137?

3. Clarity in lines 327-331 in reference to the results shown in Supplementary Figure 8, (e), in the SIVmacVif antagonism of cpzA3H, only the W73A CBFbeta mutant interferes with Vif-mediated degradation (in this combination the R52A mutation does not impair). These distinctions are important to support the authors conclusions of viral lineage comparisons.

4. It is perhaps not too surprising that HIV-1 Vif and HIV-2 Vif antagonism of A3H results in the same lysine being ubiquitinated. Lysine selection during ubiquitination is constrained by structural accessibility (as shown in this submission) but also primary sequence and motifs contributing to local context required by the critical enzymes.

Reviewer #3

(Remarks to the Author)
(General comments)
The manuscript Skorupka et al. reported a structural study of the endemic virus HIV-2 Vif in complex with an anti-HIV restriction factor APOBEC3H from chimpanzee using single-particle cryo-EM. This work reports a structure of HIV-2 Vif/A3H/CBF-b/EloBC complex with A3H in previously reported dsRNA-mediated dimer form. This is an excellent follow up study of their recent publication on HIV-1 Vif-cpzA3H complex (reference 35). The interface between A3H and HIV-2 Vif is novel and distinct from that of HIV-1 Vif with any of APOBEC3s reported in recent years. This is also the first study in the field reporting the structure of HIV-2 Vif, which has only 23% sequence identity with that of HIV-1. The resolution of the cryo-EM map is solid, and density reasonably supports most atomic models. Extensive functional study well validates the observed Vif-A3H interface, ubiquitination sites, and direct interface with CBF-b. Thus, scientific novelty is high and overall quality of the manuscript meets the standard of Nature Communications. That said, I would encourage the authors to review the following points.

(Specific points)
The major critic may be that chimpanzee A3H - HIV-2 Vif is not natural pair. Authors should explicitly discuss this when introducing the cpzA3H as a surrogate of human homolog. In authors' previous study (reference 35), they observed dimeric form of A3H-VCBC complex. Was similar dimeric complex of A3H-V2CBC observed in this study? Were there any potential 2D or 3D classes representing the dimeric complex? This needs to be discussed.

(Minor points)
1. In the main density map, there is extra density near A3H-B, which likely represents another Vif bound to this A3H protomer. In Fig.1a, it would be more appropriate to color this extra density differently from the density representing A3H-B. Any comments or insights on this observation would be helpful for readers.
2. The sequence alignment in Fig. 4a should be revised in a way that the two sequences are displayed once (they are the same sequence) with residues of interest highlighted.
3. In line 243, " 4 and 5" is likely the mislabeling of " 3 and 4". Please double check.
4. In Fig.7, the extended model of the HIV-2 Vif-Cul5 E3 ligase complex does not contain E2 ubiquitin conjugating enzyme that is recruited by ARIH2. I understand there is no existing experimental structure of such complex. To display the location of ubiquitin and its orientation in this model, it may be reasonable to model E2 enzyme, such as UBE2L3. Additionally, ARIH1/2 proteins take a few distinct intermediate states. It would be appropriate to mention which intermediate states of ARIH1 was used to generate Ub-loaded ARIH2 model.
5. The HIV-2 Vif has a long insertion sequence between the last two short helices compared to HIV-1 Vif. Is this region entirely disordered?
6. In Supplementary Fig.1, values in X and Y axes in most chromatography panels are missing. In X-axis labels, "retention time (mL)" should be either "retention time (min)" or "retention volume (mL)". Please mention the type of columns in the legend as well.

Version 2:

Reviewer comments:

Reviewer #1

(Remarks to the Author)
I think the revision has properly addressed my concerns.

Reviewer #2

(Remarks to the Author)
The manuscript, Divergent interfaces, convergent outcome: structural basis of HIV-2 Vif antagonism of APOBEC3H, reveals, through a variety of experiments, the critical structural interface and essential molecular interactions between the host restriction factor (A3H) and its viral antagonist (HIV-2 Vif). Unexpectedly, the analysis also uncovers a novel important interface between CBFbeta and A3H that has not been previously described. The biological significance of the findings is highlighted and verified in functional assays performed in tissue culture.
My comments for improvement of the manuscript were mostly satisfied in the submitted revised manuscript. However, one important point remains to be experimentally addressed; the author revision in currently insufficient. The correction will be quick and strengthen the presentation of the results.

Original reviewer suggestion:
5. In Figure 5g, it is somewhat debatable whether the C73A/D100A substitution confers significant protection from degradation. These bands could be quantitated with ImageQuant if the argument is important enough to the conclusions.

Author response:
We thank the reviewer for this suggestion. We have quantified the immunoblot bands in Figure 5g by densitometry analysis and display the percentage of cpzA3H expression levels directly beneath the Western blot image. Wild-type cpzA3H in the absence of HIV2 Vif (NMC716) was set to 100% as a reference. The quantification reveals that the C73A/D100A double substitution confers modest protection from Vif-mediated degradation, with 20–30% of cpzA3H remaining compared to wild-type.

Revised manuscript comment:
The analysis the authors have completed does not yet allow the comparison of protein expression/degradation for the experiment shown in Figure 5g. To properly quantify the bands to allow this comparison, densitometry analysis must be completed not just on the wild-type cpzA3H bands but also the beta-tubulin bands (loading control). The beta-tubulin expression serves as the normalization control for the wild-type cpzA3H expression. The densitometry analysis should be calculated as a ratio of wild-type cpzA3H expression: beta-tubulin expression (normalization control). After these calculations it is then appropriate to set the wild-type cpzA3H in the absence of HIV2 Vif (NMC716) to 100% as a reference.

It is not clear from the revised manuscript that this analysis was completed properly as there is no mention of the use of beta-tubulin as a normalization control in the procedure to address reviewer comments in the revised work. I strongly encourage the authors to correct the analysis to strengthen confidence in their findings.

Reviewer #3

(Remarks to the Author)
All my concerns in the last version have been addressed by the authors in the present submission, and I recommend the manuscript for publication. I noticed a similar study by Niu et al (doi: s41467-026-75045-1) have been published recently and therefore this article may be considered for accelerated publication.

**Open Access** This Peer Review File is licensed under a Creative Commons Attribution 4.0 International License, which permits use, sharing, adaptation, distribution and reproduction in any medium or format, as long as you give appropriate credit to the original author(s) and the source, provide a link to the Creative Commons license, and indicate if changes were made.
In cases where reviewers are anonymous, credit should be given to 'Anonymous Referee' and the source.
The images or other third party material in this Peer Review File are included in the article’s Creative Commons license, unless indicated otherwise in a credit line to the material. If material is not included in the article’s Creative Commons license and your intended use is not permitted by statutory regulation or exceeds the permitted use, you will need to obtain permission directly from the copyright holder.

To view a copy of this license, visit https://creativecommons.org/licenses/by/4.0/

We thank all reviewers for the thorough and constructive assessment and for recognizing the strengths of our study. We have carefully addressed all specific concerns raised below, and believe the revised manuscript is substantially strengthened by these revisions.

**REVIEWER COMMENTS**

Reviewer #1 (Remarks to the Author):

*Major concerns*
*1. Physiological relevance to human A3H variants requires clearer treatment*
*The structural work relies primarily on chimpanzee A3H (cpzA3H), while the manuscript extrapolates conclusions to human A3H haplotypes. Because human A3H is highly polymorphic and exhibits major haplotype-dependent differences in stability and Vif susceptibility, the manuscript should more clearly define the limitations of extrapolating from cpzA3H to human A3H. Additional functional testing using at least one or two human A3H haplotypes, particularly haplotype II, would substantially strengthen the translational relevance of the study. If such experiments are not feasible, the Discussion should more explicitly acknowledge this limitation.*

We thank the reviewer for this important comment. To address the physiological relevance of our structural findings to human A3H, we have included a sequence alignment of cpzA3H and human A3H haplotype II (**Supplementary Figure 6a**), which reveals that the two proteins differ at only nine residues. Critically, 20 out of 21 residues that directly contact HIV-2 Vif are conserved between cpzA3H and human A3H hapII, with the single exception of E178 in cpzA3H, which is substituted by D178 in human hapII — a conservative substitution that preserves the negative charge and is unlikely to alter the interaction with R18 of A3H (**Supplementary Fig. 6b**). These observations support the structural and functional relevance of our cpzA3H-based model to human A3H hapII, and we have added this discussion to the revised manuscript to more clearly define the translational scope of our findings.

**Functional degradation data for human A3H hapII with VIF W49A/W51A mutants:** Consistent with the high conservation of interface residues, HIV-2 Vif efficiently degrades human A3H haplotype II, and W49A/W51A substitutions markedly attenuate this degradation, directly validating the translational relevance of our structural model. We have added this result as **Supplementary Fig. 6c**.

**Acknowledge limitations:** We have added following sentence in Discussion. “Although our structure is based on cpzA3H, 20 of 21 Vif-contacting residues are conserved in human A3H haplotype II. Consistently, HIV-2 Vif degrades human A3H haplotype II in a CBFβ- and CUL5-dependent manner, and W49A/W51A substitutions markedly attenuate this degradation (**Supplementary Fig. 6c**), validating the translational relevance of our model. Nonetheless, haplotype-specific differences in protein stability, subcellular localization, and RNA binding may modulate degradation efficiency, and future studies incorporating all human A3H haplotypes will be needed to fully define the basis of haplotype-dependent Vif susceptibility.”

2. *The proposed ubiquitination geometry appears largely model-driven*
*The manuscript proposes a structural model explaining how A3H K50 is positioned relative to the ubiquitination machinery. However, the full CUL5–RBX2-containing assembly appears to be substantially lower resolution and partially dependent on rigid-body fitting and homology modeling. The manuscript should more explicitly distinguish experimentally resolved structural features from inferred modeling. The Discussion and figure legends should clarify the uncertainty associated with the proposed ubiquitination geometry and avoid overstating mechanistic precision that is not directly supported by the cryo-EM density.*

We thank the reviewer for this important critique. We fully agree that a clear distinction between experimentally resolved features and model-derived inferences is essential for scientific rigor. Our high-resolution cryo-EM density defines the HIV-2 Vif–cpzA3H–CBFβ–EloBC core complex at 3.08 Å resolution, within which the positioning of cpzA3H K50 relative to Vif and EloBC is directly supported by experimental density. The extended CUL5–RBX2-containing assembly, however, was modeled by rigid-body fitting of available structures (PDB: 7ONI and 7B5M) into lower-resolution density, and the proposed ubiquitination geometry of K50 therefore represents a structurally informed model rather than an experimentally resolved mechanism. We have revised the figure legend and Discussion to explicitly distinguish these two levels of structural evidence and to more carefully qualify the mechanistic interpretation of the ubiquitination geometry.

*Additional technical and presentation issues*
*1. Even though the structure reveals a unique CBFβ/A3H interface in the HIV-2 complex, the HIV-1 A3F complexes also show CBFβ contacts (as discussed by the authors). Maybe tone down like: previously unobserved for A3H, or structurally distinct from HIV-1 A3F interactions, etc, to be on the safe side.*

We thank the reviewer for this suggestion and have revised the text as follows. “The structure reveals the molecular basis of HIV-2 Vif–A3H recognition and uncovers a direct CBFβ–A3H interface that is not present in previously characterized HIV-1 Vif-A3H complexes.”

*2. I cannot quantitatively assess complex formation shifts in Supplementary Figure 1.*

We thank the reviewer for this comment. We have revised the x-axis label in **Supplementary Figure 1** to indicate retention volume (mL), which should allow quantitative assessment of the complex formation shifts.

*3. The manuscript occasionally blurs HIV-2 vs SIVmac conclusions and discussion. I feel it would be easier for me to read it if there is a clearer distinction between directly tested systems (HIV-2 NMC716 with cpzA3H) and the inferred evolutionary generalizations.*

We thank the reviewer for this suggestion and have revised the text as follows. "Within this framework, HIV-2 Vif appears, based on evolutionary inference, to retain ancestral features of SIVsmm Vif that are broadly compatible with primate APOBEC3 proteins, …."

4. *The manuscript discusses severe preferred orientation that was partially alleviated by addition of nCUL5, although nCUL5 density is absent from the final reconstruction. Additional directional FSC or 3D FSC analysis would help readers evaluate any residual anisotropy in the final map.*

We thank the reviewer for this suggestion. We have added a 3D FSC analysis panel to **Supplementary Figure 3** showing residual anisotropy in the final reconstruction. Although nCUL5 supplementation was critical for improving particle orientation distribution during data collection, nCUL5 itself is absent from the final map. The 3D FSC analysis confirms that residual anisotropy is minimal and does not compromise the resolution or interpretability of the cpzA3H–V2CBC interface, the primary focus of our structural analysis.

5. *Supplementary Figure 3 includes unexplained additional density proposed to correspond to a second V2CBC complex. The authors should clarify the basis for this interpretation and explain why the density was excluded from the final reconstruction.*

We thank the reviewer for this observation. The additional density in **Supplementary Figure 3a** corresponds to a second V2CBC complex bound to the distal cpzA3H protomer (cpzA3HB), supported by 2D classification analysis now shown in panel f. Particles containing two V2CBC copies were not excluded from the final reconstruction; their inclusion helped overcome the preferred orientation bias and improved data quality. However, the second V2CBC copy could not be reliably resolved to high resolution despite extensive processing. The additional density is displayed in the map (shown in blue in panel a) to provide transparency about its presence, while the final atomic model is restricted to the well-resolved primary V2CBC copy.

6. *The representative 2D class averages in Supplementary Figure 2 are difficult to evaluate because of their small size and low contrast. Enlarged representative class averages would improve presentation quality*.

We thank the reviewer for this suggestion. We have updated **Supplementary Fig. 2** to include higher-contrast representative 2D class averages that more clearly illustrate the major structural features and domain organization of the cpzA3H–V2CBC complex.

7. Some biochemical controls appear incomplete. For example, several Vif mutants in Supplementary Figure 5 appear to produce A3H levels above the no-Vif control. It would be important to show Vif expression levels for all mutants to distinguish direct functional effects from altered protein stability or expression.

We thank the reviewer for this suggestion. We have added a Western blot in **Supplementary Figure 5b** quantifying HIV-2 Vif expression levels for all mutants, normalized to β-tubulin. Most mutants maintained >90% of wild-type Vif expression levels; only the W74A mutant showed reduced expression at ~80%. These results demonstrate

that the observed impairment of A3H degradation reflects direct functional defects in Vif rather than secondary effects on Vif protein expression or stability.

*8. The statement that W7 forms an intermolecular β-sheet with CBFβ and that W13/W22 stabilize a hydrophobic core is plausible but not sufficiently illustrated in the current figures. A close-up structural panel with corresponding density would strengthen this point.*

We thank the reviewer for this suggestion. We have added **Supplementary Fig. 5c**, a close-up structural panel of the HIV-2 Vif N-terminal tryptophan cluster with cryo-EM density displayed as a transparent mesh. The panel illustrates how W7 engages CBFβ I55 and V86, W13 contacts CBFβ L64 and Vif V87/L89, and W22 stabilizes the hydrophobic core between Vif α-helices α1 and α6. These interactions collectively stabilize the N-terminal Vif–CBFβ interface, consistent with the substantial reduction in cpzA3H degradation observed upon alanine substitution of these residues in HiBiT assays.

*Reviewer #2 (Remarks to the Author):*

*Primary suggestions:*

*1. The highlighting of the important interface between CBFbeta and A3H is a unique part of this submission (as noted by the authors in line 430) and, as such, I would recommend mentioning that finding in the abstract. The important binding events extend beyond Vif and A3 and this is an interesting result that ought to be clear to the reader from the outset!*

We thank the reviewer for this suggestion and have revised the abstract to highlight the novel CBFβ–cpzA3H interface as a key finding of this study.

*2. In line 133, the phrase "…residues within the BC-box motif…" should be modified slightly because not all residues within this BC box contribute to functional Vif activity.*

We thank the reviewer for this suggestion and have revised the text as follows. "In addition, BC-box residues L148, F150, and L153 contributed to activity, consistent with their established role in EloB/C recruitment."

*3. The importance of W49 and W51 in Vif antagonism of A3 family members has been previously described in different experimental settings. In particular, I think the 2014 publication from the Pathak lab ought to be cited somewhere in the manuscript:*

We thank the reviewer for this suggestion and have added text and this reference as follows. "This result is consistent with biochemical findings by Smith *et al*., who identified these residues as critical determinants of HIV-2 Vif-induced degradation of human A3H haplotype II[46]."

*4. Figure 5f results from a meticulous and well-planned experiment. However, the degradation of cpzA3H in the absence of transfected CBFbeta is glaring. While the authors do offer a reasonable explanation for the observation in the figure legend, I would recommend moving the explanation to the main body of the narrative to ensure clarity for the reader.*

We thank the reviewer for this suggestion and have added text as follows. "The degradation of cpzA3H observed in lane 2 (**Fig. 5f**) indicates that endogenous CBFβ is sufficient to support HIV-2 Vif–induced degradation."

5. In Figure 5g, it is somewhat debatable whether the C73A/D100A substitution confers significant protection from degradation. These bands could be quantitated with ImageQuant if the argument is important enough to the conclusions.

We thank the reviewer for this suggestion. We have quantified the immunoblot bands in **Figure 5g** by densitometry analysis and display the percentage of cpzA3H expression levels directly beneath the Western blot image. Wild-type cpzA3H in the absence of HIV-2 Vif (NMC716) was set to 100% as a reference. The quantification reveals that the C73A/D100A double substitution confers modest protection from Vif-mediated degradation, with 20–30% of cpzA3H remaining compared to wild-type.

Minor suggestions:

*1. In the abstract, lines 31-32, "…HIV-2 and HIV-1 Vif both target A3H lysine 50…", as well as line 367, "…HIV-2 Vif primarily targets K50…" could be worded better for clarity. The Vif protein targets A3H for degradation by establishing a direct interaction that results in the accessibility of lysine 50. Vif does not really target an individual amino acid as the authors elegantly show with their extensive cryo-EM structure work.*

We thank the reviewer for this suggestion and have revised the text as follows.
Abstract, "Despite these structural differences, lysine 50 of cpzA3H is the shared ubiquitination target of both HIV-1- and HIV-2 Vif-induced degradation, revealing convergent function achieved through divergent structural solutions."
Line 367, "…, establishing K50 as the primary ubiquitination site for HIV-2 Vif-induced degradation. Together, these data demonstrate that K50 is the principal ubiquitination site for HIV-2 Vif-induced cpzA3H degradation, consistent with previous findings that K50 and K51 are the preferred ubiquitination sites for HIV-1 Vif-induced A3H degradation."

*2. What is the (functional/structural) relevance of the hydrophobic core described in line 137?*

We thank the reviewer for this comment and have revised text as follows. "W7 engages CBFβ I55 and V86, and W13 engages CBFβ L64 and Vif V87 and L89, through hydrophobic interactions essential for intermolecular β-sheet formation. W22 contributes to a hydrophobic core stabilizing α-helix α1 and α-helix α6 on the β-sheets of HIV-2 Vif."
In addition, we have added **Supplementary Fig. 5c**, a close-up structural panel of the HIV-2 Vif N-terminal tryptophan cluster with cryo-EM density displayed as a transparent mesh.

*3. Clarity in lines 327-331 in reference to the results shown in Supplementary Figure 8, (e), in the SIVmacVif antagonism of cpzA3H, only the W73A CBFbeta mutant interferes with Vif-mediated degradation (in this combination the R52A mutation does not impair). These distinctions are important to support the authors conclusions of viral lineage comparisons.*

We thank the reviewer for this suggestion and have revised the text as follows. "Furthermore, CBFβ R52A and W73A substitutions impaired HIV-2 ROD10-mediated degradation of cpzA3H (**Supplementary Fig. 8d**), and W73A — but not R52A — rescued cpzA3H from SIVmac-induced degradation (**Supplementary Fig. 8e**), indicating that these interactions are partly conserved across HIV-2 and SIVmac lineages."

*4. It is perhaps not too surprising that HIV-1 Vif and HIV-2 Vif antagonism of A3H results in the same lysine being ubiquitinated. Lysine selection during ubiquitination is constrained by structural accessibility (as shown in this submission) but also primary sequence and motifs contributing to local context required by the critical enzymes.*

We thank the reviewer for this observation and agree that Lys50's positioning on an exposed loop ensures accessibility to the E2–E3 ubiquitination machinery, consistent with its conserved role as the primary ubiquitin acceptor site.

Reviewer #3 (Remarks to the Author):

*(General comments)*
*The manuscript Skorupka et al. reported a structural study of the endemic virus HIV-2 Vif in complex with an anti-HIV restriction factor APOBEC3H from chimpanzee using single-particle cryo-EM. This work reports a structure of HIV-2 Vif/A3H/CBF-b/EloBC complex with A3H in previously reported dsRNA-mediated dimer form. This is an excellent follow up study of their recent publication on HIV-1 Vif-cpzA3H complex (reference 35). The interface between A3H and HIV-2 Vif is novel and distinct from that of HIV-1 Vif with any of APOBEC3s reported in recent years. This is also the first study in the field reporting the structure of HIV-2 Vif, which has only 23% sequence identity with that of HIV-1. The resolution of the cryo-EM map is solid, and density reasonably supports most atomic models. Extensive functional study well validates the observed Vif-A3H interface, ubiquitination sites, and direct interface with CBF-b. Thus, scientific novelty is high and overall quality of the manuscript meets the standard of Nature Communications. That said, I would encourage the authors to review the following points.*

*(Specific points)*
*The major critic may be that chimpanzee A3H - HIV-2 Vif is not natural pair. Authors should explicitly discuss this when introducing the cpzA3H as a surrogate of human homolog. In authors' previous study (reference 35), they observed dimeric form of A3H-VCBC complex. Was similar dimeric complex of A3H-V2CBC observed in this study? Were there any potential 2D or 3D classes representing the dimeric complex? This*

*needs to be discussed.*
We thank the reviewer for these important points.

**Regarding cpzA3H as a surrogate for human A3H:** We acknowledge that cpzA3H-HIV-2 Vif is not a natural pair. We have explicitly discussed this limitation and addressed it by demonstrating that HIV-2 Vif efficiently degrades human A3H haplotype II in a CBFβ- and CUL5-dependent manner, with conserved key contact residues (W49/W51), validating the translational relevance of our cpzA3H-based model (see also our response to Reviewer 1's similar comment).

**Regarding monomer vs. dimer complex formation:** In contrast to the dimeric cpzA3H–VCBC complex observed in our previous study, the cpzA3H–V2CBC complex is monomeric, as determined by size-exclusion chromatography and confirmed by cryo-EM structural analysis (added to Results section). This distinction likely reflects mechanistic differences in how HIV-1 and HIV-2 Vif engage A3H substrates and has been discussed in the revised manuscript.

(Minor points)
1. In the main density map, there is extra density near A3H-B, which likely represents another Vif bound to this A3H protomer. In Fig.1a, it would be more appropriate to color this extra density differently from the density representing A3H-B. Any comments or insights on this observation would be helpful for readers.

We thank the reviewer for this comment, which was also raised by Reviewer 1 (see our detailed response there). Briefly, the additional density in **Supplementary Figure 3a** corresponds to a second V2CBC complex bound to the distal cpzA3H protomer (cpzA3HB), supported by 2D classification analysis (panel f). Particles with two V2CBC copies were retained in the final reconstruction to mitigate preferred orientation bias, but the second copy could not be reliably resolved at high resolution. This density is displayed in the map (blue, panel a), while the atomic model is restricted to the well-resolved primary V2CBC copy (**Fig. 1**).

2. *The sequence alignment in Fig. 4a should be revised in a way that the two sequences are displayed once (they are the same sequence) with residues of interest highlighted.*

We thank the reviewer for this suggestion. We have revised **Figure 4a** to display the cpzA3H sequence once, with residues contacting HIV-1 Vif and HIV-2 Vif highlighted in their respective colors.

*3. In line 243, "$\alpha$4 and $\alpha$5" is likely the mislabeling of "$\alpha$3 and $\alpha$4". Please double check.*

We thank the reviewer for this suggestion and have revised the text.

4. *In Fig.7, the extended model of the HIV-2 Vif-Cul5 E3 ligase complex does not contain E2 ubiquitin conjugating enzyme that is recruited by ARIH2. I understand there is no existing experimental structure of such complex. To display the location of ubiquitin and its orientation in this model, it may be reasonable to model E2 enzyme, such as*

*UBE2L3. Additionally, ARIH1/2 proteins take a few distinct intermediate states. It would be appropriate to mention which intermediate states of ARIH1 was used to generate Ub-loaded ARIH2 model.*

We thank the reviewer for this suggestion. We have added **Supplementary Figure 11**, which depicts ARIH2 bound to UBE2L3 in Transition State 1, representing the early stages of ubiquitin transfer prior to ubiquitination of $A3H_A$. In comparison, **Figure 7** illustrates the ubiquitin-charged ARIH2 in Transition State 2, representing the catalytic step of ubiquitin transfer to $A3H_A$.

*5. The HIV-2 Vif has a long insertion sequence between the last two short helices compared to HIV-1 Vif. Is this region entirely disordered?*

Yes, the long insertion sequence in HIV-2 Vif positioned between the final two short helices exhibits high conformational flexibility and lacks interpretable cryo-EM density, preventing reliable structural modeling of this region.

6. *In Supplementary Fig.1, values in X and Y axes in most chromatography panels are missing. In X-axis labels, “retention time (mL)” should be either “retention time (min)” or “retention volume (mL)”. Please mention the type of columns in the legend as well.*

We thank the reviewer for this observation. We have updated **Supplementary Fig. 1**, with the X-axis now correctly labeled as "retention volume (mL)" across all chromatography panels. Additionally, the column types used for each complex have been specified in the figure legend.

We thank the reviewers for their thorough and constructive assessment and for recognizing the strengths of our study. We have carefully addressed all remaining concerns, and believe the revised manuscript is substantially strengthened as a result.

**REVIEWER COMMENTS**

*Reviewer #2*

*Revised manuscript comment:*
*The analysis the authors have completed does not yet allow the comparison of protein expression/degradation for the experiment shown in Figure 5g. To properly quantify the bands to allow this comparison, densitometry analysis must be completed not just on the wild-type cpzA3H bands but also the beta-tubulin bands (loading control). The beta-tubulin expression serves as the normalization control for the wild-type cpzA3H expression. The densitometry analysis should be calculated as a ratio of wild-type cpzA3H expression: beta-tubulin expression (normalization control). After these calculations it is then appropriate to set the wild-type cpzA3H in the absence of HIV2 Vif (NMC716) to 100% as a reference.*

*It is not clear from the revised manuscript that this analysis was completed properly as there is no mention of the use of beta-tubulin as a normalization control in the procedure to address reviewer comments in the revised work. I strongly encourage the authors to correct the analysis to strengthen confidence in their findings.*

We thank the reviewer for this important suggestion. We have performed densitometry on both cpzA3H and β-tubulin bands, normalizing cpzA3H signal to the corresponding β-tubulin signal to control for loading variability. Normalized cpzA3H levels are expressed as a percentage relative to wild-type cpzA3H in the absence of HIV-2 Vif (NMC716, set to 100%). These values are now displayed below each lane in the updated Figure 5g.
